# Cost-effectiveness of tuberculosis evaluation and treatment of newly-arrived immigrants

**DOI:** 10.1186/1471-2458-6-157

**Published:** 2006-06-19

**Authors:** Travis C Porco, Bryan Lewis, Elliot Marseille, Jennifer Grinsdale, Jennifer M Flood, Sarah E Royce

**Affiliations:** 1 California Department of Health Services, Tuberculosis Control Branch,850 Marina Bay Parkway, Building P, Second Floor, Richmond, CA 94804, USA; 2 University of California, Berkeley, Center for Infectious Disease Preparedness, 1918 University Way, Berkeley, CA 94704, USA; 3 Institute for Health Policy Studies, University of California, San Francisco, USA; 4 San Francisco Department of Public Health, San Francisco General Hospital, Ward 94,1001 Potrero Avenue, San Francisco, CA 94110, USA

## Abstract

**Background:**

Immigrants to the U.S. are required to undergo overseas screening for tuberculosis (TB), but the value of evaluation and treatment following entry to the U.S. is not well understood. We determined the cost-effectiveness of domestic follow-up of immigrants identified as tuberculosis suspects through overseas screening.

**Methods:**

Using a stochastic simulation for tuberculosis reactivation, transmission, and follow-up for a hypothetical cohort of 1000 individuals, we calculated the incremental cost-effectiveness of follow-up and evaluation interventions. We utilized published literature, California Reports of Verified Cases of Tuberculosis (RVCTs), demographic estimates from the California Department of Finance, Medicare reimbursement, and Medi-Cal reimbursement rates. Our target population was legal immigrants to the United States, our time horizon is twenty years, and our perspective was that of all domestic health-care payers. We examined the intervention to offer latent tuberculosis therapy to infected individuals, to increase the yield of domestic evaluation, and to increase the starting and completion rates of LTBI therapy with INH (isoniazid). Our outcome measures were the number of cases averted, the number of deaths averted, the incremental dollar cost (year 2004), and the number of quality-adjusted life-years saved.

**Results:**

Domestic follow-up of B-notification patients, including LTBI treatment for latently infected individuals, is highly cost-effective, and at times, cost-saving. B-notification follow-up in California would reduce the number of new tuberculosis cases by about 6–26 per year (out of a total of approximately 3000). Sensitivity analysis revealed that domestic follow-up remains cost-effective when the hepatitis rates due to INH therapy are over fifteen times our best estimates, when at least 0.4 percent of patients have active disease and when hospitalization of cases detected through domestic follow-up is no less likely than hospitalization of passively detected cases.

**Conclusion:**

While the current immigration screening program is unlikely to result in a large change in case rates, domestic follow-up of B-notification patients, including LTBI treatment, is highly cost-effective. If as many as three percent of screened individuals have active TB, and early detection reduces the rate of hospitalization, net savings may be expected.

## Background

The global tuberculosis (TB) epidemic strongly influences the incidence of TB within California, as evidenced by the occurrence of 75 percent of California TB cases in 2002 in persons born outside the U.S. [1]. Screening of foreign-born persons is often recommended [2] to detect active TB at an earlier stage. Such screening and evaluation may also identify individuals with latent TB infection (LTBI), for whom therapy could prevent future disease [3].

Federal law requires immigrants to the U.S. to undergo an overseas examination for TB (and other conditions) [4]. Individuals are screened with chest radiographs (and sputum smears for acid fast bacteria, if indicated) to identify potentially infectious individuals who are barred from entry into the U.S. Those with active TB who are sputum smear-negative, or who have inactive TB, receive a B-notification; such individuals are instructed to report to a local health jurisdiction within 30 days after entry. In California (1992 to 1996), three and one half percent of such persons with a B-notification were reported to have active TB within one year of arrival [5]. Upon domestic evaluation, individuals without active disease may fall into one of three categories (using the American Thoracic Society classes [6]): TB0 (no evidence of infection), TB2 (evidence of infection, but no evidence of disease), or TB4 (stable radiographic abnormalities suggestive of TB together with evidence of TB infection, and negative bacteriologic studies) [7]. Individuals in classes TB2 and TB4 are eligible for LTBI therapy, unless already treated. Studies of domestic follow-up have found that compared to recently-arrived TB cases in California without B-notification, those with a B-notification were reported with TB sooner after their arrival in the U.S. [5], suggesting that domestic follow-up of B-notification patients is detecting cases sooner than they would have been detected passively. Cases with a B-notification were less likely to have smear-positive pulmonary disease (unsurprisingly, since smear-positive individuals are not assigned to class B and cannot legally enter the U.S. until treatment has resulted in smear conversion); nevertheless, some B-notification patients have smear-positive disease upon domestic follow-up.

Is domestic follow-up by local health jurisdictions of patients identified as suspected cases overseas a good public health investment, and if so, how should those resources be most efficiently invested? To date, the only U.S. cost-effectiveness study focused on the savings that result from excluding cases from entry into the U.S., not on the yield of domestic follow-up [8]. In this report, we examine the cost-effectiveness [9-15] of the domestic follow-up of B-notification patients by determining the number of TB cases prevented, the number of deaths among persons with TB averted, and the number of quality-adjusted life-years (QALYs) saved, for each dollar invested. We first consider only the costs and benefits resulting from domestic evaluation and active case-finding, i.e., the earlier detection of cases of active TB disease. We then determine the additional costs and additional benefits of therapy of latent TB infection (LTBI) for suitable persons identified by domestic evaluation. Finally, we determine the most cost-effective means to improve the yield of the program.

## Methods

### Overview

**Figure 1:**
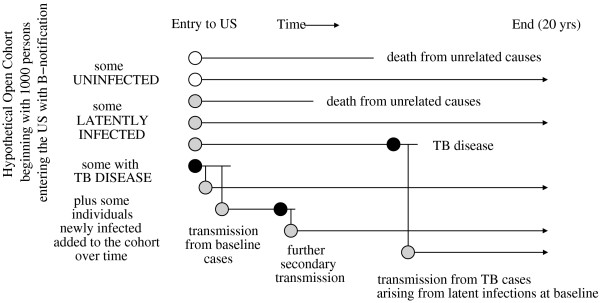
**B-notification cohort**. A B-notification cohort in the absence of treatment is followed for twenty years. Uninfected individuals are symbolized by white circles, latently infected individuals (TB2 and TB4) by gray circles, and cases of active disease by black circles. Vertical lines symbolize transmission of *Mycobacterium tuberculosis*.

We determined how domestic B-notification follow-up would change the expected dollar cost, the number of incident cases of TB, the number of deaths among individuals with TB, and the number of QALYs lost, by simulating the natural history of a hypothetical cohort of 1000 B-notification patients and their infected contacts for 20 years after entry (Figure 1). Individuals at the baseline may have active disease, may be latently infected (and possibly develop active disease during the 20-year analytic horizon), or may even be uninfected (because radiologic screening is not highly specific [4,7]). Individuals with active TB may infect new individuals, who are then added to the simulated cohort. Domestic B-notification followup would detect individuals at the baseline who have active disease, and such earlier detection would reduce costs and transmission [16-18]. Screening for active disease also identifies individuals in class TB2 or TB4; additional resources spent on their LTBI therapy reduce future TB [19], but may cause hepatitis in some individuals [20]. We neglect the incidence of infection among those uninfected at baseline (because this is unrelated to B-notification follow-up), although some uninfected individuals will be misclassified as being latently infected (and thus incur costs and some adverse health outcomes). We used an individual-based stochastic simulation (for example, see [21]) based on a natural history model of tuberculosis, e.g. [22-26]. Because the model structure reflected individual variability in TB progression, for each scenario we averaged the results of 10 000 replications of the model to determine the model outputs we report.

### Perspective

We adhered to the reference case scenario recommended by the Panel on Cost-effectiveness in Health and Medicine [27], except that (1) we took a domestic all-payers perspective rather than a societal perspective (we excluded indirect costs of lost productivity, lost wages, and time spent seeking health care), and (2) we excluded the costs of the overseas screening examination, as well as the benefits of overseas treatment of the smear-positive cases discovered before immigration.

We chose this perspective to provide guidance to local health jurisdictions who may need to decide how much to invest in follow-up of new immigrants with tuberculosis B-notifications. Given that overseas screening is currently mandated for new immigrants to the United States and that the expense of such screening is already being incurred, what benefits may be gained through appropriate follow-up domestically? Or equivalently, what opportunities are missed should we fail to follow-up on those patients who have already been identified?

All costs and health effects were discounted at three percent per year.

### Cohort at baseline

The age-distribution was determined from B-notifications in the period 2001 to 2003 reported in California. We conservatively assumed that zero to seven percent of the cohort at the beginning of the study period (i.e., at baseline) have active TB (conservatively assuming only seven and one half percent of these to be smear-positive). One study reported three and one half percent of B-notification immigrants were reported to have active TB within one year after arrival in the U.S., but 13 percent were smear positive [5], though higher rates of active disease have been found in studies where more refugees were present [7,28]. We also assumed that 11 percent are in class TB2, and 36 percent are persons in class TB4 who are eligible for LTBI therapy. The remainder are assumed to require no therapy (because they are either uninfected, or they are former active cases of TB who received adequate therapy for their TB disease). These proportions were derived from unpublished local California health department data, B-notification surveillance forms provided to the State of California by local health jurisdictions, and the literature [7].

### LTBI therapy

According to American Thoracic Society guidelines [29], we assume that all B-notification patients in class TB2, regardless of age, are candidates for therapy to prevent progression to active TB disease, because LTBI is not detected or treated in the major countries of origin. We assumed that providers prescribed nine months of isoniazid (INH), and that a complete course has a 70 percent efficacy rate in preventing progression to active TB disease [19]; we also assumed that some individuals would fail to complete 9 months of therapy, but would receive some benefit from partial completion (see Appendix [Additional file 1] for details).

We used estimates from the literature for the frequency of INH-induced hepatitis, both fatal and non-fatal, as well as the probability of non-hepatitis side effects [20,30-34]. We assume that costs of nonfatal hepatitis include three additional physician visits and three sets of liver function tests. For ten percent of hepatitis patients requiring hospitalization, we assumed a seven day hospitalization [35]. Non-hepatitis side effects were assumed to require one additional outpatient MD visit.

### Natural history

Individuals with latent infection were assumed to develop TB according to a declining function as described in other models [9,10,36,37]; we assumed a given declining risk for all TB2 patients, and a higher risk for TB4s. Newly infected individuals are assumed to be at higher risk of disease for two years, after which time they are assumed to be equivalent to other TB2s. Our choice of declining exponential risk was based on results from a cohort of recent immigrants from Southeast Asia to Australia; this cohort may have included recently exposed individuals at higher risk for progression (which would have increased the average risk of the cohort), but individuals with abnormal chest X-ray were specifically excluded [38-40].

While, as discussed before, we assumed that most individuals with B-notification that have active disease upon entry to the U.S. are found to be smear-negative, we assume that some fraction of smear-negative individuals will progress to smear-positive if mortality or diagnosis does not occur. Smear-positive cases are assumed to have higher hospitalization costs and to be more contagious [16,17]. We also assumed that actively detected cases have lowered hospitalization rates, even controlling for sputum smear status [16,17]. Finally, we calculate race- and age-specific TB mortality rates using Report of Verified Case of Tuberculosis forms (RVCTs) submitted by California local health departments from 1996 to 2000, the most recent years with complete outcomes.

Our model also includes some risk of transmission from within the cohort, and the resulting higher risk of progression among those newly infected individuals. We assume that each smear-positive case may produce new infections over time at a rate of from 3 to 13 per year [41]; please see the Appendix for complete details ([see Additional file 1]).

### Program performance

Program performance is the local health department's ability to maintain patient participation, i.e., to minimize attrition as the patient presents for and completes the evaluation, starts LTBI therapy, and completes LTBI therapy. We assumed a range of attrition rates for the cohort based on unpublished B-notification follow-up rates in California local health departments and the literature (see Appendix [Additional file 1] for details).

Costs were varied according to the increase in units of medical services (for example, tuberculin skin tests and chest radiographs) needed to serve the larger number of clients retained. We assumed that improvements in the fraction evaluated could be achieved by letters, phone calls, or home visits [42], and that improvements in the fraction completing LTBI therapy couldbe achieved by placing some individuals on directly observed preventive therapy [43].

### Health state utilities

Health state utilities as found in the literature [9,44-48] were used to calculate the QALYs gained and lost through the therapy for LTBI and treatment of active TB disease. In the absence of final results from ongoing studies that have demonstrated the feasibility of determining health-state utilities for tuberculosis patients [49,50], we used the best estimates available from the literature and from previous cost-effectiveness analyses. The mean duration patients experienced the different health states were determined using California-specific data and the assumption that providers were following ATS guidelines.

Specifically, we assumed that the total lost QALYs for fatal tuberculosis during hospitalization was 0.043, for nonfatal tuberculosis during hospitalization was 0.021, and for outpatient tuberculosis was 0.05. We assumed that the health state utility for side effects of INH sufficient to warrant discontinuation (other than hepatitis) was 0.9, for outpatient INH hepatitis was 0.735, for hepatitis under hospitalization was 0.12, and for tuberculosis prior to treatment was 0.9. For the base case, we assumed no disutility for INH pill-taking in the absence of side-effects sufficient to warrant discontinuation. The rationale for these choices, and the uncertainty analytic ranges, are discussed further in the Appendix [see Additional file 1].

### Costs

Selected costs were estimated using a variety of sources. We assumed a standard set of medical services for diagnosis and treatment; these costs are summarized in Table 1. Medical costs were adjusted to 2004 using the Medical Care component of the Consumer Price Index. We used Medicare physician fee schedules where-ever possible (using a weighted average over the nine Medicare geographic pricing regions in California based on the number of tuberculosis cases seen in that region of California in 2004).

Otherwise, we used average nationwide allowed charges (Medicare Part B) [51]; average nationwide figures, however, may underestimate California costs to some extent. Where Medicare Part B charges were not available, we used current Medi-Cal reimbursement rates (for the cost of DOT visits and nurse prescription refill visits; see Appendix [Additional file 1] for further discussion). Table 1 excludes costs of MDR-TB (since INH preventive therapy cannot prevent it, such cases would occur with or without the program), as well as HIV/TB coinfection (since individuals with HIV infection are currently excluded from legal immigration, and we assume the risk of infection between the overseas examination and immigration is small).

Little information is available to determine the cost of nurse symptom review or prescription refill visits; current Medi-Cal reimbursement rates are approximately in agreement with one-half hour of staff RN time (using a recent salary survey [52]). By contrast, our use of Medi-Cal reimbursement rates to estimate DOT costs may be conservative, underestimating the true costs; for instance, the inflated cost of one DOT visit on average (assuming five per week, and using the Medical care component of the CPI) would be approximately $24 in 2004 dollars [53], higher than the $19 we assumed per visit. However, we assumed a fixed fraction of patients on DOT, and did not need to adjust the expected costs of tuberculosis for the cost-saving features of the use of DOT [51,54].

### Base case scenario, and uncertainty and sensitivity analyses

Selected parameters for our base case scenario are given in Table 2; a complete discussion is given in the Appendix [see Additional file 1]. We chose plausible ranges for uncertain parameters in the model, repeating analyses with values chosen from a uniform distribution within the ranges, and conducted univariate sensitivity analyses.

## Results

### Base case scenario

For our base case scenario, we found that domestic B-notification follow-up consisting of evaluation and case treatment is highly cost effective. Including therapy for LTBI as well as treatment of active cases, we found that the program yielded 8 net QALYs, yielded $25 000 in net savings, and prevented 4 cases of TB. We determined the number of quality-adjusted life-years saved, the net cost, the number of cases prevented, and the number of deaths averted during 20 years as a result of treating only active cases (class TB3), as well as offering LTBI treatment for individuals in ATS class TB4 and ATS class TB2. Summary results from our base case scenario are shown in the first line of Table 6. The active case finding component was estimated to have prevented approximately 0.5 new cases of TB; the treatment of individuals in class TB4 to have prevented approximately 3.1 new cases, and finally the treatment of individuals in class TB2 to have prevented approximately 0.6 new cases. In this scenario, the costs of the evaluation are offset by the potential savings due to earlier case finding and prevention of disease by the use of LTBI therapy. Because the evaluation costs were attributed to the active case finding component of the program, the treatment of TB4s was in fact cost saving, while the treatment of TB2s remained highly cost effective (yielding QALYs at a rate of approximately $4 400 per QALY, and preventing cases at a cost of $4 700 per case prevented as well).

### Sensitivity analysis

In Tables 3, 4, 5, we vary three program performance parameters: the evaluation rate, the starting rate, and the completion rate, keeping all other parameters the same as in the base-case scenario (we assume, however, no targeted use of directly observed preventive therapy is needed, and assume outreach costs for evaluation are limited to the cost of sending letters). The tables show the costs and benefits for programs of different performance, assuming the same variable cost structure. In Table 3, we present the number of QALYs saved, net costs, cases averted, and deaths with TB averted that are attributable to the active case finding component. In Table 4, we show the results attributable to treatment of individuals in ATS class TB4, and in Table 5, that attributable to treatment of individuals in ATS class TB2 (i.e., incremental results for the decision to treat individuals in class TB4 and class TB2, respectively). The upper and middle values of the evaluation rates, starting rates, and completion rates were based on unpublished data from selected California local health jurisdictions.

In Table 6, we present the results of univariate sensitivity analysis. Line One of the table shows results from the base case scenario; we show the cost-effectiveness of evaluation and treating active cases only, the incremental cost-effectiveness of treating TB4s, and finally the overall cost-effectiveness of evaluation and therapy for active cases, individuals in class TB4, and individuals in class TB2.

Assuming a mean passive treatment delay of 100 days, or a screening delay of two weeks on average following entry into the U.S., had little effect (note that this result depends on an assumption that few smear-negative individuals will become smear positive during this short time). Assuming six percent of the population are active TB cases (instead of three percent) results in far greater health benefits (cases prevented, deaths averted, QALYs saved) and far greater cost savings; in our base case, the evaluation costs were almost exactly offset by savings due to treatment and interrupting transmission, and when there are more cases to be found, the cost savings considerably exceed the evaluation costs. Increasing the number of new infections per year that a case can cause to 16 per year for a smear-positive case resulted in more QALYs saved and in cost savings (line 5). We varied the four hospitalization rates as shown in the Table; higher hospitalization rates for actively found cases and lower hospitalization rates for passively found cases all resulted (as expected) in increased net costs. In the least cost-effective of these scenarios, the overall cost-effectiveness ratio was still approximately $20 000 per QALY saved, well below the commonly-cited willingness-to-pay threshold of $50 000 per QALY (and the cost per case prevented was approximately $35 000). We also found that a 20 percent increase in all costs other than tuberculosis hospitalization costs essentially eliminated the predicted overall net cost-savings without changing the finding that the domestic B-notification follow-up is cost-effective. On the other hand, a 20 percent increase in hospitalization costs renders each program component cost-saving on average, since each prevented case saves more money. When we assumed that the reactivation rates for individuals in class TB4 were 430 per 100 000 person-years (instead of 600 per 100 000), the treatment of TB4s became approximately cost-neutral, but still saved QALYs and prevented cases. Similarly, we examined the cost-effectiveness of treating individuals classified as TB2 in their evaluation (not shown in Table 6). In the base case scenario, the reactivation rate was 217 per 100 000 person years [38-40] (see Appendix [Additional file 1] for details), and the treatment of individuals identified as TB2 was still cost-effective as discussed earlier. With an incidence rate of 100 per 100 000 person-years, however, the expected gain in QALYs was only 0.3, the cost-effectiveness ratio is approximately $27 000 per QALY, and the net cost to prevent one case is approximately $30 000.

Because of the potential importance of INH-induced hepatitis, we also examined the consequences of assuming that the levels of hepatitis risk were higher than we assumed for the base case. We considered the case of treating individuals in ATS class 2, who were at the lowest risk for tuberculosis progression. We found that when the base case rates were multiplied by a factor of approximately eighteen or more, preventive therapy led to a net loss of QALYs; preventive therapy yields QALYs at a cost of less than $50 000 per QALY provided the hepatitis rates are no greater than approximately fifteen times the values we assumed in the base case. Although all deaths due to INH-related hepatitis are, in principle, attributable to the intervention, while not all deaths with tuberculosis can be assumed to be attributable to tuberculosis, the results of this sensitivity analysis would appear to support the robustness of findings that the benefits of tuberculosis prevention, on the whole, outweigh the adverse outcomes due to isoniazid. Because of the importance of the fraction of individuals screened who have active disease, we performed a threshold analysis for this parameter by repeating the base case, but varying only the fraction with active disease (and fitting a linear model to the simulation results). These findings suggest that the intervention becomes net cost saving when the fraction of active cases is approximately 2.7 percent or greater, but that the intervention remains cost-effective at the willingness-to-pay threshold of $50 000 provided the active case fraction is greater than 0.4 percent (0.004). The estimated cost-effectiveness ratio for various levels of the active case fraction are provided in Table 8.

We also examined different values of the proportion of individuals in ATS class TB4 and in ATS class TB2. Barring special circumstances, individuals in ATS class TB2 should not receive a tuberculosis B-notification under current policies, since such individuals have normal chest X-rays, but as discussed above, a fraction of tuberculosis B-notifications nonetheless have been found to be in ATS class 2. In Table 7, we assumed that the fraction of active cases was either 1.5% or 3%, that the fraction of individuals in ATS class TB4 varied from 30% to 75%, and that the fraction of individuals in ATS class TB2 who were not in ATS classes 3 or 4 varied from 15% to45% (providing the total fraction was less than 1.0, as shown). We present the average net cost and QALYs saved by evaluation and treatment of TB3s, and then the further incremental net cost and incremental number of QALYs saved by treatment of TB2s and TB4s, and finally the aggregate net cost and number of QALYs saved by evaluation and treatment of active cases, TB2s, and TB4s (last two columns). In this table, we averaged the results of 1000 random simulations and present mean values only (omitting the standard errors for the sake of brevity). Table 7 shows that for a given active case fraction, an increasing fraction of individuals in ATS class TB4 corresponds both to increased costs (owing to the greater evaluation costs of individuals with abnormal chest X-ray) and to increased opportunity for prevention of TB. The least cost-effective scenario we examined had 1.5% active cases, but 75% TB4s; the cost per QALY saved was approximately $21 000, and the cost per case prevented was approximately $29 000.

### Resource allocation

What do these results imply for improvement of public health performance based on cost-effectiveness? We used our model to compare the cost-effectiveness of improvement of evaluation rates with offering LTBI therapy to individuals of class TB2 or TB4, and with efforts to improve starting or completion rates among those populations offered LTBI therapy. We applied the data from a study of efforts to improve B-notification evaluation rates in Santa Clara County, California [42] using letters, phone calls, and home visits.

Our results are shown in Table 9. Beginning with a purely passive program which only treated active cases without actively seeking them, we found that the highest first priority was a low-cost measure to improve evaluation rates (sending letters), which yielded approximately 3 QALYs and a net savings of approximately $10 000. Once this was implemented, the best step was to begin treating individuals identified as in class TB4, yielding approximately 3 QALYs at a net savings of $11 000; treating TB4s accomplishes more once the improvements in evaluation are in place. The next step is to improve starting rates of LTBI therapy for TB4s now that it has been decided to offer therapy. After this, treatment of TB2s is the next best step. Finally, further improvements in evaluation up to home visits continue to add a small additional benefit. Directly observed preventive therapy, however, does not appear cost-effective (yielding QALYs at a cost of in excess of $100 000) in this population (we have assumed that there is no HIV infection in this population, and we have not modeled any other risk factors for progression). The rankings in Table 9 are somewhat sensitive to the assumed outreach efficiencies, fraction of active cases and TB4s, and other parameters and may thus vary somewhat from place to place. However, improvement in evaluation rates, low cost efforts to improve starting rates, and offering therapy to individuals (both TB2 and TB4) identified through domestic B-notification remain highly cost-effective to cost-saving for a wide range of parameter values.

### Overall benefits

What are the benefits from domestic B-notification follow-up to California as a whole? With approximately 3 700 B-notifications for California in 2002, the scenarios in Tables 3, 4, 5 suggest that domestic follow-up for one year would prevent approximately 6–26 cases per year over time (see Appendix [Additional file 1] for details), with slight estimated annual savings of approximately $67 000–$170 000 (though these values are highly sensitive to changes in the fraction of active cases found and the hospitalization costs).

## Discussion

We found that evaluation and treatment of active cases for new immigrants with tuberculosis B-notifications is highly cost-effective, yielding quality-adjusted life years at approximate cost neutrality. We assumed, however, that actively detected cases are substantially less likely to be hospitalized than passively detected cases [16]. We also found that offering LTBI therapy for individuals identified as having LTBI through their domestic B-notification evaluation is highly cost-effective as well, and is in fact cost saving on average for individuals in class TB4. We found that, provided monitoring maintains relatively low levels of INH-related hepatitis [20], the benefits of LTBI therapy outweigh the potential harm due to hepatitis (as other studies have found). Our results also suggest that program performance may be improved in a cost-effective manner, and that the highest priorities should be LTBI therapy for individuals in ATS class TB4 and improvement of evaluation rates. Importantly, treatment of individuals in class TB2 was still cost-effective, though our results suggest that routine use of directly-observed therapy for LTBI cases (with no othermedical risk factors) may fall short of common willingness-to-pay thresholds. It is important, however, to realize that this conclusion applies only to domestic B-notification patients at low risk of HIV infection, and that we assumed no other risk factors for tuberculosis progression. For populations with HIV or other risk factors for progression, directly observed preventive therapy may be cost-effective [13].

However, despite the cost-effectiveness of domestic B-notification follow-up, we cannot expect such follow-up to decrease the number of overall California TB cases by more than approximately one percent. While our cost-effectiveness arguments imply that domestic B-notification is valuable, it simply reaches too few people to have a large effect on the overall case rate. Other measures, such as enhanced screening and evaluation of other foreign-born populations (such as undocumented residents or individuals on short-term visas) or extending the overseas screening to attempt toidentify individuals with LTBI [2], would be needed to cause a larger effect on the total number of cases.

Several limitations apply to our findings. Our findings were derived from a transmission model of TB transmission, case-detection, and reactivation; we attempted to make conservative assumptions and to use a wide range for many parameters. For many of the costs of the model, accurate estimates are not available; we utilized Medicare reimbursement rates wherever possible, and constructed a highly conservative cost accounting for tuberculosis medical costs; we nevertheless found that domestic follow-up was cost-saving or highly cost-effective – the true cost savings may be higher than we found. We also used expected years of life in the absence of available estimates of quality-adjusted life expectancy, and we attributed all deaths of TB patients to tuberculosis itself. Sensitivity analysis, however, suggests that these assumptions do not change our conclusion that domestic B-notification follow-up is cost-effective under widely accepted willingness-to-pay standards (e.g. $50 000 per QALY gained). Furthermore, note that our model did not include lost productivity, which would have improved the cost-effectiveness of the interventions examined. Also, because individuals with HIV infection are generally barred from immigration, our model did not address the cost-effectiveness of TB prevention among the HIV-infected. Also, we did not address whether individuals beyond a certain age should or should not receive LTBI therapy; our results only demonstrate net cost-effectiveness aggregating over age groups. Uncertainty in the benefits of active case finding plays a major role in shaping the variability in our findings, and more information would be needed to precisely characterize the medical and fiscal benefits of B-notification follow-up. Finally, parameter estimation uncertainty (especially for the fraction of active cases among those screened, the progression rate from smear-negative to smear-positive, hospitalization costs (for actively and passively diagnosed cases) and the rate of reactivation of LTBI) limits theprecision of our results. It is also important to note that decisions based on aggregating QALYs across individuals may not yield results in agreement with the expressed social preferences of most people, a finding which, in particular, may occur when small increments of QALYs aggregated across many people outweigh large increments in QALYs for a few, e.g. [55,56]. This circumstance arises in our model when small decrements in utility due to INH pill-taking for all individuals undergoing preventive therapy may accumulate to outweigh the tuberculosis deaths prevented (as seen in Table 6). However, for the base case, the number of net QALYs saved is largely the result of tuberculosis cases (and resulting deaths) prevented (hepatitis, and other side effects being relatively small contributors), and serves to allow us to compare our proposed interventions to commonly-accepted willingness-to-pay standards.

Our analytic perspective was chosen to provide guidance to local health jurisdictions who may be faced with the choice of how much to invest in domestic follow-up of individuals already identified through tuberculosis B-notification. This choice of analytic perspective, however, led us to exclude the costs of the overseas screening itself, both to the immigrant and to the U.S. (since we assume the program as mandated and that these costs are in any event paid), as well as any overseas benefits of the overseas screening itself (such as earlier detection of smear-positive cases). Thus, our results have no bearing on the cost-effectiveness of overseas screening as such [11,12,14,57] or of the current U.S. screening policies in particular, but serve rather to emphasize the value of domestic follow-up of patients who have already been screened – given the policy as it is currently being implemented.

## Conclusion

Our findings indicate that domestic B-notification follow-up is a cost-effective intervention that is warranted under commonly-cited willingness-to-pay thresholds; domestic B-notification benefits the patients served as well as the general community in a cost-effective manner. While most of the benefits result from the active case-finding component, it is also cost-effective to treat latent TB infection. Large changes in the case burden, however, cannot be expected from domestic B-notification follow-up under current conditions. Nevertheless, our conclusions support augmented investment in the domestic follow-up in B-notification, and suggest that the program is not only cost-effective, but may actually be cost-saving.

## Authors' contributions

All authors contributed to the design and implementation of the study. The authors declare no competing financial interests. TP, BL, and EM developed the model and conducted parameter evaluation, and TP drafted the manuscript. JG and JF conducted parameter evaluation. SR conceived of the study and participated in its design and coordination, and contributed to parameter evaluation and drafting the manuscript. All authors read and approved the final manuscript.

## Pre-publication history

The pre-publication history for this paper can be accessed here:



## Supplementary Material

Additional file 1 Appendix Click here for file

## Figures and Tables

**Table 1:** Program Cost Data. Program cost data derived from Medi-Cal reimbursements or other sources (column 1). All costs given in U.S. dollars, adjusted to year 2004 using the Medical Care component of the U.S. Consumer Price Index (U.S. Bureau of Labor Statistics). Note 1: For each procedure for which Medicare charges were computed, we obtained charges for all nine regions in California for which Medicare calculates a geographic price adjustment factor. We then computed a weighted average based on the year 2004 tuberculosis case count for the nine regions. Note 2: Year 2003 Medicare Part B average national allowed charges (BESS) Note 3: Assuming a standard regimen of isoniazid, pyrazinamide, rifampin and ethambutol for two months, followed by isoniazid and rifampin for four months, assuming a 65 kg adult. Note 4: weighted average of the average daily hospitalization cost for Los Angeles, San Diego, San Francisco [58,59], converted to 2004 dollars; weights derived from cases reported in 1995 [60] (during the CDC Cost of Hospitalization Study). Note 5: Inflated to 2004 dollars by the medical Consumer Price Index. Note 6: These items inflated using the all-items Consumer Price Index.

Variable	Cost	Source
New patient doctor visit	111.50	Medicare, 2004, Note 1
Established patient visit	61.25	Medicare, 2004, Note 1
Tuberculin skin test	11.78	Medicare, 2004, Note 1
Initial chest X-ray	39.68	Medicare, 2004, Note 1
Sputum collection	17.80	Medicare, 2004, Note 1
Specimen concentration	9.33	Medicare Part B, Note 2
AFB smear	7.50	Medicare Part B, Note 2
Sputum culture	14.42	Medicare Part B, Note 2
Sensitivity testing	7.38	Medicare Part B, Note 2
Mycobacterial identification	15.29	Medicare Part B, Note 2
Isoniazid, 100 tablets	8.90	Medicaid Federal Upper Limit price
Chemotherapy for active TB	1118	Drug prices Red Book, 2004; ATS recommendations; Note 3
Nurse assessment, 1/4 hr	8.40	Medi-Cal
Liver function tests	11.42	Medicare Part B, Note 2
TB hospitalization episode	25 834	Note 4; based on CDC unpublished data
Hepatitis hospital stay	10 662	Note 5; [61]
Complete blood count	9.04	Medicare Part B, Note 2
Renal function panel	12.13	Medicare Part B, Note 2
Directly observed therapy (DOT)	19.23	Medi-Cal
Disease control investigation	387.02	[51,52]; Appendix
Letter mailed	10.80	[42]; Note 6
Phone call (community worker)	13.24	[42]; Note 6
Home visit (community worker)	39.57	[42]; Note 6

**Table 2:** Base case scenario parameters. Selected parameters for cost-effectiveness of California tuberculosis B-notifications. Full details and additional parameters are given in the Appendix [see Additional file 1]. Note 1: California TB surveillance for individuals from major countries of origin, 2002. Note 2: one minus the relative risk of disease given completion of LTBI therapy relative to no therapy, assuming isoniazid-sensitive infection. Note 3: assuming no false positives due to prior BCG vaccination. Note 4: rate per 100 000 person-years. See Appendix [Additional file 1] for further details and complete references.

**Description**	**Values**
**Mean age (Note 1)**	53.3
**INH resistance**	0.129
**Drug-induced hepatitis rates**	0.0008 to 0.0028
**Efficacy of completed regimen (Note 2)**	70%
**Mean time to passive diagnosis**	74 days
**TST sensitivity**	93%
**TST specificity (Note 3)**	99%
**Probability of fast progression **to TB after infection	0.05
Probability of finding a TB contact	80%
**Fraction of individuals with B-notification**	
with active disease	3%
in class TB4	60%
in class TB2	22%
**Fraction of**	
baseline active cases smear positive	7.5%
TB4s eligible for LTBI therapy	63%
**Hospitalization probability**	
actively detected smear-positive	0.35
actively detected smear-negative	0.081
passively detected smear-positive	0.66
passively detected smear-negative	0.51
**Transmission (new infections per year)**	
smear-positive source case	8
smear-negative source case	0.8
**Attrition cascade for domestic follow-up after B-notification**	
Evaluation rate	60%
Starting rate, LTBI therapy	80%
Completion rate, LTBI therapy	75%
**Reactivation rate at baseline**, Note 4	
class TB4	600
class TB2	217

**Table 3:** Program performance. The incremental cost-effectiveness of evaluation and treatment of disease among a hypothetical cohort of 1 000 B-notification patients followed for 20 years. This table presents the outcomes for the active case finding component of the program, and lists the number of QALYs averted, the net cost, the number of cases averted, and the difference in the number of deaths among individuals with tuberculosis with and without active case finding, for three levels of the evaluation rate. All outcomes are discounted at a rate of three percent per year. Each row is computed by averaging 10 000 replications of a cohort of 1 000 individuals each, over a 20-year period; standard errors are given in parentheses.

Evaluation Rate	Starting Rate	Completion Rate	QALYs Saved	Net costs (1000s)	Cases Averted	TB Deaths Averted
45%	-	-	1.9 (0.09)	-11 (0.5)	0.18 (0.02)	0.059 (0.003)
65%	-	-	2.9 (0.08)	-16 (0.5)	0.26 (0.02)	0.089 (0.004)
85%	-	-	3.9 (0.08)	-22 (0.5)	0.38 (0.02)	0.12 (0.003)

**Table 4:** Program performance. The incremental cost-effectiveness of evaluation and treatment of disease among a hypothetical cohort of 1000 B-notification patients followed for 20 years. The table presents the outcomes of a decision to treat individuals in ATS class TB4, listing the number of QALYs averted, the net cost, the number of cases averted, and the difference in the number of deaths among individuals with tuberculosis with and without the program, for various levels of the evaluation rate, the starting rate, and the completion rate. All outcomes are discounted at a rate of three percent per year. Each row is computed by averaging 10 000 replications of a cohort of 1 000 individuals each, over a 20-year period; standard errors are given in parentheses.

Evaluation Rate	Starting Rate	Completion Rate	QALYs Saved	Net costs (1000s)	Cases Averted	TB Deaths Averted
45%	35%	30%	1.1 (0.1)	-3.2 (1)	0.79 (0.06)	0.039 (0.005)
45%	35%	55%	0.63 (0.2)	-2.4 (0.7)	0.95 (0.05)	0.018 (0.008)
45%	35%	75%	0.79 (0.1)	-1.9 (0.9)	1 (0.05)	0.032 (0.007)
45%	45%	30%	0.85 (0.2)	-3.6 (0.7)	0.97 (0.04)	0.028 (0.009)
45%	45%	55%	1.3 (0.3)	-3.8 (0.9)	1.1 (0.05)	0.048 (0.01)
45%	45%	75%	1.4 (0.2)	-4.5 (0.8)	1.4 (0.05)	0.058 (0.01)
45%	80%	30%	1.8 (0.1)	-6.6 (1)	1.7 (0.05)	0.07 (0.006)
45%	80%	55%	2.5 (0.2)	-6.7 (1)	2.1 (0.04)	0.088 (0.009)
45%	80%	75%	2.4 (0.2)	-6.9 (1)	2.4 (0.06)	0.09 (0.01)

65%	35%	30%	1.3 (0.2)	-5.2 (1)	1.2 (0.04)	0.049 (0.009)
65%	35%	55%	1.7 (0.2)	-3.4 (1)	1.3 (0.05)	0.065 (0.009)
65%	35%	75%	1.4 (0.1)	-3.3 (0.7)	1.5 (0.03)	0.048 (0.009)
65%	45%	30%	1 (0.2)	-6.1 (2)	1.4 (0.08)	0.04 (0.007)
65%	45%	55%	2.1 (0.2)	-6.1 (0.7)	1.8 (0.04)	0.077 (0.01)
65%	45%	75%	2.2 (0.2)	-5.5 (0.9)	1.9 (0.03)	0.086 (0.009)
65%	80%	30%	2.5 (0.2)	-11 (2)	2.6 (0.07)	0.095 (0.008)
65%	80%	55%	3 (0.2)	-10 (0.8)	3 (0.04)	0.11 (0.01)
65%	80%	75%	3.5 (0.1)	-8.3 (0.6)	3.4 (0.03)	0.13 (0.007)

85%	35%	30%	1.7 (0.2)	-5.6 (1)	1.4 (0.06)	0.065 (0.007)
85%	35%	55%	2.2 (0.2)	-7.4 (1)	1.8 (0.04)	0.089 (0.008)
85%	35%	75%	2.2 (0.3)	-5.6 (0.7)	1.9 (0.04)	0.087 (0.01)
85%	45%	30%	2 (0.2)	-8.1 (0.9)	1.9 (0.04)	0.074 (0.01)
85%	45%	55%	2.3 (0.2)	-8 (1)	2.4 (0.04)	0.091 (0.008)
85%	45%	75%	2.5 (0.2)	-6.6 (0.8)	2.6 (0.04)	0.093 (0.01)
85%	80%	30%	3.7 (0.2)	-14 (1)	3.4 (0.05)	0.14 (0.01)
85%	80%	55%	4.1 (0.1)	-13 (0.7)	3.9 (0.03)	0.15 (0.009)
85%	80%	75%	4.5 (0.2)	-12 (0.9)	4.4 (0.04)	0.16 (0.008)

**Table 5:** Program performance. The incremental cost-effectiveness of evaluation and treatment of disease among a hypothetical cohort of 1000 B-notification patients followed for 20 years. The table presents the outcomes of a decision to treat individuals in ATS class TB2, listing the number of QALYs averted, the net cost, the number of cases averted, and the difference in the number of deaths among individuals with tuberculosis with and without the program, for various levels of the evaluation rate, the starting rate, and the completion rate. All outcomes are discounted at a rate of three percent per year. Each row is computed by averaging 10 000 replications of a cohort of 1 000 individuals each, over a 20-year period; standard errors are given in parentheses.

Evaluation Rate	Starting Rate	Completion Rate	QALYs saved	Net costs (1000s)	Cases Averted	TB Deaths Averted
45%	35%	30%	0.12 (0.08)	0.022 (0.4)	0.2 (0.02)	0.0073 (0.005)
45%	35%	55%	0.22 (0.1)	0.43 (0.4)	0.2 (0.02)	0.011 (0.006)
45%	35%	75%	0.25 (0.07)	0.09 (0.5)	0.23 (0.02)	0.013 (0.004)
45%	45%	30%	0.17 (0.08)	0.34 (0.2)	0.2 (0.02)	0.013 (0.004)
45%	45%	55%	0.32 (0.06)	0.86 (0.4)	0.24 (0.02)	0.016 (0.002)
45%	45%	75%	0.23 (0.06)	1.1 (0.6)	0.28 (0.02)	0.011 (0.003)
45%	80%	30%	0.35 (0.1)	1.4 (0.5)	0.33 (0.02)	0.014 (0.006)
45%	80%	55%	0.42 (0.1)	2.3 (0.5)	0.4 (0.02)	0.018 (0.005)
45%	80%	75%	0.55 (0.06)	2.4 (0.4)	0.45 (0.02)	0.025 (0.004)

65%	35%	30%	0.21 (0.07)	0.24 (0.4)	0.21 (0.02)	0.01 (0.003)
65%	35%	55%	0.1 (0.07)	1.3 (0.4)	0.25 (0.02)	0.0025 (0.004)
65%	35%	75%	0.28 (0.07)	1.9 (0.2)	0.29 (0.01)	0.016 (0.004)
65%	45%	30%	0.26 (0.08)	1.1 (0.4)	0.27 (0.01)	0.01 (0.005)
65%	45%	55%	0.44 (0.07)	0.87 (0.4)	0.36 (0.03)	0.019 (0.003)
65%	45%	75%	0.62 (0.06)	1.7 (0.4)	0.42 (0.02)	0.026 (0.004)
65%	80%	30%	0.51 (0.04)	1.6 (0.3)	0.49 (0.01)	0.024 (0.003)
65%	80%	55%	0.72 (0.09)	1.9 (0.5)	0.64 (0.03)	0.035 (0.004)
65%	80%	75%	0.64 (0.07)	3.7 (0.3)	0.69 (0.03)	0.028 (0.003)

85%	35%	30%	0.34 (0.08)	1 (0.4)	0.3 (0.02)	0.015 (0.004)
85%	35%	55%	0.34 (0.1)	1.4 (0.5)	0.32 (0.02)	0.014 (0.004)
85%	35%	75%	0.29 (0.07)	2.4 (0.5)	0.37 (0.02)	0.013 (0.004)
85%	45%	30%	0.37 (0.07)	1 (0.5)	0.36 (0.02)	0.016 (0.004)
85%	45%	55%	0.36 (0.05)	2.4 (0.6)	0.43 (0.02)	0.015 (0.003)
85%	45%	75%	0.49 (0.08)	3 (0.6)	0.46 (0.03)	0.022 (0.005)
85%	80%	30%	0.59 (0.07)	2 (0.3)	0.66 (0.02)	0.026 (0.002)
85%	80%	55%	0.77 (0.09)	3.9 (0.5)	0.79 (0.02)	0.034 (0.003)
85%	80%	75%	0.85 (0.06)	5.3 (0.4)	0.85 (0.02)	0.036 (0.003)

**Table 6:** Sensitivity analysis. Univariate sensitivity analysis of base case scenario (based on 10 000 replications per line). All QALYs and costs are discounted at three percent per year (unless indicated otherwise). Line One gives results for the base case scenario; in each other line, one parameter is varied from the Base Case value (column 2) to a new value (column 3). All costs are given in $1 000 2004 U.S. dollars. Negative costs represent savings. Standard errors are given in parentheses. Note 1: new infections per year caused by a smear positive case. Note 2: rate per 100 000 person-years.

Parameter varied	Value		Active cases		Add TB4s		Overall (all)	
	Base	New	QALYs	Cost	QALYs	Cost	QALYs	Cost
None	-	-	4.1 (0.3)	-17 (1.6)	2.9 (0.1)	-10 (0.7)	7.7 (0.3)	-25 (1.5)

Passive treatment delay (days)	74	100	5.8 (0.2)	-14 (1.4)	3.7 (0.2)	-12 (1)	10 (0.3)	-22 (1.7)
Screening delay (days)	0	14	4.4 (0.3)	-17 (1.4)	3.2 (0.2)	-11 (1.1)	8.1 (0.2)	-25 (1.7)
Fraction of active cases	3%	6%	8.1 (0.3)	-290 (2.2)	3.1 (0.2)	-11 (0.7)	12 (0.4)	-290 (2.5)
Baseline smear-positive rate	7.5%	15%	5.2 (0.3)	-9.6 (0.9)	3.3 (0.3)	-11 (0.8)	9.3 (0.4)	-18 (0.9)
Transmission rate (Note 1)	8	16	4.2 (0.3)	-22 (1.6)	3.4 (0.2)	-12 (1)	8.3 (0.3)	-33 (1.6)
Hospitalization rates								
Actively detect, smear pos.	0.35	0.66	4.1 (0.2)	0.8 (1)	3.6 (0.2)	-11 (1)	8.3 (0.3)	-7.2 (1.6)
Passively detect, smear pos.	0.66	0.33	3.8 (0.13)	+8.3 (1.7)	3.4 (0.2)	-1.0 (0.7)	+7.9 (0.3)	+12 (2.3)
Actively detect, smear neg.	0.081	0.162	4.1 (0.2)	+27 (0.7)	3 (0.18)	-8.6 (0.6)	7.7 (0.3)	+22 (1.2)
Passively detect, smear neg.	0.51	0.255	3.5 (0.3)	+140 (0.6)	3.2 (0.15)	1.5 (0.8)	7.3 (0.3)	+150 (1)
Reactivation rates, Note 2								
TB4s	600	430	4 (0.3)	-16 (1.3)	2.5 (0.15)	1.0 (0.7)	7.1 (0.3)	-12 (2)
Cost multipliers								
Hospitalization costs	same	+20%	4.1 (0.3)	-67 (1.5)	3 (0.13)	-16 (0.9)	7.8 (0.3)	-83 (1.5)
Costs, excluding hospital	same	+20%	3.4 (0.18)	+14 (0.9)	3.5 (0.2)	-4.6 (1.1)	7.7 (0.3)	+14 (1.6)
Selected costs								
Nurse refill visit	16.80	8.40	3.7 (0.2)	-17 (1.1)	3.3 (0.13)	-26 (0.8)	7.5 (0.3)	-43 (1.4)
DOT visit	19.23	25.00	4.5 (0.2)	-16 (1.2)	3.0 (0.2)	-11 (1)	7.8 (0.4)	-24 (1.8)
TST specificity	0.99	0.875	4.1 (0.2)	-14 (1.1)	3.0 (0.2)	-11 (0.9)	7.6 (0.3)	-21 (1.2)
Fraction INH resistant	0.13	0.2	3.9 (0.3)	-14 (1)	2.3 (0.2)	-5.3 (0.8)	6.7 (0.4)	-16 (0.9)
Risk multiplier, severe hepatitis	1	3	3.9 (0.3)	-16 (0.9)	3.0 (0.15)	-5.4 (0.5)	7.6 (0.3)	-17 (1)
Selected disutilities								
Hepatitis hospitalization	0.4	0.9	4.3 (0.3)	-15 (1.5)	3.1 (0.2)	-10 (0.9)	8.0 (0.3)	-23 (1.6)
Outpatient hepatitis	0.265	0.5	4.3 (0.2)	-16 (1.3)	3.1 (0.17)	-9.9 (1.1)	8.0 (0.3)	-24 (2)
Other INH side-effects	0.1	0.2	3.9 (0.2)	-15 (1)	2.9 (0.13)	-10 (0.7)	7.4 (0.2)	-22 (1.2)
Untreated TB	0.1	0.2	4.8 (0.19)	-15 (1)	3.6 (0.15)	-10 (0.9)	9.0 (0.3)	-22 (1.5)
Lost QALYs, INH one month	0	0.01	4.5 (0.3)	-15 (1.1)	-15 (0.2)	-9.9 (0.8)	-16 (0.4)	-22 (0.9)
Disutility multipliers								
TB hospitalization	1	0.5	4.0 (0.19)	-16 (1.4)	3.3 (0.1)	-10 (1)	7.9 (0.2)	-24 (1.3)
Outpatient TB	1	0.5	4.2 (0.3)	-16 (1.3)	3.4 (0.2)	-8 (0.7)	8.3 (0.3)	-21 (1.6)
Discount rate per year	0.03	0.05	3.2 (0.15)	-13 (1)	2.4 (0.13)	-6.9 (1.2)	5.9 (0.2)	-16 (1.6)

TST sensitivity	0.93	0.95	4.2 (0.2)	-17 (1.3)	3.4 (0.15)	-11 (1)	8.3 (0.3)	-25 (1.7)
TST specificity	0.99	0.95						

**Table 7:** Fraction latently infected. Bivariate sensitivity analysis of base case scenario, varying the percentage of individuals in ATS classes TB3 (active cases), TB4, and TB2. The results reported are the average values for 1 000 replications, with standard errors given in parentheses. All QALYs and costs are discounted at three percent per year; costs are listed in thousands of 2004 U.S. dollars, and negative costs represent savings. All parameters are the same as in the base case scenario unless otherwise indicated; the results shown assume that treatment will be undertaken on individuals in ATS class TB2 and TB4 whenever possible.

Percent Active TB	Percent TB4	Percent TB2	QALYs saved	Net cost	Cases averted
1.5	30	15	4.3 (0.2)	66 (1.1)	2.3 (0.05)
1.5	30	30	4.4 (0.3)	67 (1.5)	2.7 (0.07)
1.5	30	45	5.4 (0.3)	72 (1.2)	3.0 (0.07)
1.5	45	15	5.0 (0.4)	88 (1.3)	3.0 (0.09)
1.5	45	30	5.2 (0.4)	90 (1.2)	3.4 (0.07)
1.5	45	45	6.0 (0.2)	94 (0.9)	3.9 (0.06)
1.5	60	15	5.7 (0.4)	110 (1.1)	3.8 (0.07)
1.5	60	30	6.2 (0.3)	120 (1.6)	4.0 (0.07)
1.5	75	15	6.4 (0.3)	130 (1.9)	4.5 (0.11)

3.0	30	15	6.2 (0.2)	-67 (1.2)	2.5 (0.06)
3.0	30	30	6.4 (0.3)	-64 (1.4)	2.8 (0.06)
3.0	30	45	7.0 (0.2)	-64 (1.2)	3.3 (0.05)
3.0	45	15	6.8 (0.3)	-46 (1.9)	3.1 (0.06)
3.0	45	30	7.2 (0.4)	-42 (1.4)	3.6 (0.11)
3.0	45	45	7.5 (0.4)	-40 (1.1)	4.0 (0.07)
3.0	60	15	7.4 (0.3)	-25 (2)	4.0 (0.15)
3.0	60	30	8.5 (0.3)	-21 (1.8)	4.5 (0.1)
3.0	75	15	9.1 (0.3)	-1.4 (1.6)	4.8 (0.07)

**Table 8:** Cost-effectiveness. Estimated cost-effectiveness ratio as the fraction of active cases vary, with all parameters otherwise equal to the base case scenario. All QALYs and costs are discounted at three percent per year; costs are listed in 2004 U.S. dollars. The cost-effectiveness ratios are based on the assumption that all active cases and latently infected individuals (ATS class 2 and class 4) are eligible for treatment.

Fraction of active cases	40% TB4s		60% TB4s	
	
	Cost per QALY saved	Cost per case averted	Cost per QALY saved	Cost per case averted
0%	$72 000	$72 000	$64 000	$66 000
0.5%	$47 000	$57 000	$45 000	$53 000
1.0%	$30 000	$41 000	$31 000	$41 000
1.5%	$17 000	$27 000	$19 000	$29 000
2.0%	$7 000	$12 000	$10 000	$17 000
2.5%	cost saving	cost saving	$3000	$5 000
3.0%	cost saving	cost saving	cost saving	cost saving

**Table 9:** Program priorities. Program priorities determined by decision analysis of the base case scenario, based on considering (1) improvement of evaluation rates by letters, phone calls, and home visits, (2) offering LTBI therapy to individuals in class TB2 or class TB4, (3) improving rates of starting LTBI therapy to those it is offered to (by education and outreach), and (4) improvement of rates of completion of LTBI therapy to those it is offered to (by targeted DOPT). Beginning with a minimal program (line 1), the available options (column two) were compared. Options that produced net health benefits and cost savings were always preferred over options that produced net health benefits at a net total cost; whenever more than one option produced health benefits and cost savings, we chose the option yielding the greatest savings (other choices of decision rule are possible). Options yielding health benefits at net cost were to be ranked according to the cost-effectiveness ratio. The resulting program actions are given in the left hand column of the following row, which forms the basis for the next comparison; notice that the costs and benefits of a given course of action (such as starting TB4s on LTBI therapy) depend on previous choices (such as whether or not we have sent letters to improve evaluation rates, yielding more TB4s to treat). The parameters and assumptions of the base case are discussed in the text and Appendix [Additional file 1].

	Beginning with ...	Choose between ...	Best choice
1.	Treat only active cases; detect them only passively	(1) Offer LTBI treatment to TB2s or TB4s, or (2) send letters to improve evalua tion	Send letters (2.7 QALYs gained, $10 000 in net sav ings)
2.	Send letters; treat active cases	(1) Offer LTBI treatment to TB2s, (2) Offer LTBI treatment to TB4s, or (3) make phone calls to improve evaluation rates	Treat TB4s (3.2 QALYs gained, $11 000 in net savings)
3.	Treat active cases and TB4s; improve evaluation by letters	(1) Offer LTBI treatment to TB2s, (2) make phone calls to improve evaluation rates further, (3) improve rates of starting ther apy for TB4s, or (4) improve completion rates by DOPT	Improve starting rates (1.3 QALYs saved, $1 800 in net savings)
4.	Treat active cases and TB4s; improve evaluation rates by letters; improve starting rates	(1) send letters to improve evalua tion rates further, (2) treat TB2s, or (3) improve completion rates by DOPT	Treat TB2s (0.7 QALYs saved, $3 000 in net cost)
5.	Treat active cases, TB2s, and TB4s; improve evaluation by letters; improve rates of starting therapy	(1) Further improve evaluation rates by phone calls, or (2) improve rates of completing therapy (by targeted DOPT)	Phone calls (0.5 QALYs saved, approximately $1 000 in net savings)
6.	Treat active cases, TB4s, and TB2s; improve evaluation by letters and phone calls	(1) Further improve evaluation rates by home visits, or (2) improve rates of completing therapy by using targeted DOPT	Home visits (0.3 QALYs saved, approximately $1 000 in net cost)
7.	Treat active cases, TB4s, and TB2s; improve evaluation by letters and phone calls	(1) improve rates of completing therapy by using targeted DOPT	> $100 000 per QALY saved; no further intervention
