# Supplementary material for: Cost-effectiveness of tuberculosis evaluation and treatment of newly-arrived immigrants
Source: BMC Public Health. 2006 Jun 19;6:157. doi: 10.1186/1471-2458-6-157 (PMC1559699; doi:10.1186/1471-2458-6-157)
Supplement: Additional file 1 — Appendix [file 1471-2458-6-157-S1.pdf]

Appendix: Cost-effectiveness of Tuberculosis Evaluation and Treatment of Newly-Arrived Immigrants

Travis C. Porco, Ph.D., M.P.H.[1,2]*, Bryan Lewis, M.P.H.[1], Elliot Marseille, Dr.P.H., M.P.P.[3], Jennifer Grinsdale, M.P.H.[4], Jennifer M. Flood, M.D.[1], and Sarah E. Royce, M.D.[1]

1 California Department of Health Services, Tuberculosis Control Branch
2151 Berkeley Way, Suite 608, Berkeley, CA 94704 USA
2 University of California, Berkeley, Center for Infectious Disease Preparedness,
1918 University Dr., Berkeley, CA 94704 USA
3 Institute for Health Policy Studies, University of California San Francisco
4 San Francisco Department of Public Health, San Francisco General Hospital, Ward 94,
1001 Potrero Avenue, San Francisco, CA 94110

* To whom correspondence should be directed: Travis C. Porco, California Department of Health Services, Tuberculosis Control Branch, 2151 Berkeley Way Suite 608, Berkeley, CA 94704; email: TPorco@dhs.ca.gov; phone: (510) 540–3516; fax: (510) 540–2062.

May 10, 2006

1 Introduction

In this Appendix, we provide full details about the model structure and parameter choices we used to simulate the cost-effectiveness of domestic follow-up for new immigrants with B-notification.

2 Overview

2.1 Model structure

We modeled the progression and transmission of tuberculosis using a multistate transition model, implemented as an event-driven simulation. In our model, we follow an initial cohort of newly-arrived immigrants with B-notifications, and these individuals may be evaluated (and then may be diagnosed as active cases, or may receive therapy for latent tuberculosis infection), or may not be evaluated. Active, undiagnosed cases may transmit infection, and these newly infected individuals are added to the simulation cohort. Individuals may be in one of several tuberculosis-related classifications, and undergo transitions to other states as a result of medical and epidemiological processes (such as diagnosis, treatment, endogenous reactivation of latent infection, etc.) We also assume that individuals are classified into one of three public health categories: (A) the individual is not being (or no longer being) sought for tuberculosis evaluation, (B) the individual is being sought for domestic B-notification follow-up, and finally (C) the individual is a close contact of an active case who has not been screened or examined for active or latent tuberculosis. Our transition model is similar to models used in previous cost-effectiveness analyses[1] and for the analysis of transmission[2, 3, 4].

2.2 Outcome variables

Our outcome variables are (1) the number of cases, (2) the number of deaths of individuals with tuberculosis, (3) the expected cost, and (4) the number of Quality-Adjusted Life Years (QALYs) lost in the cohort and among individuals in chains of infections traceable to the cohort. Unfortunately, however, precise estimates of the health state utilities needed to derive QALYs for the health states of our model for our population are not available at this time; the specific choices we made are discussed below.

We discounted both costs and quality-adjusted life-years at the same rate (three to five percent) to reflect social time-preference.

2.3 Evaluation criteria

When comparing program alternatives, we ranked the alternatives by the incremental cost-effectiveness ratio, computed as the number of dollars per QALY (discounted)[5, 6]. However, whenever an intervention resulted in cost savings as well as health benefits, we assumed that any such intervention was a higher priority than an intervention for which health benefits were obtained at net cost. When two interventions both resulted in cost savings as well as health benefits, we ranked them by the incremental number of QALYs saved.

2.4 Computing benefits to California as a whole

To compute the benefits of domestic evaluation and follow-up to California as a whole, we multiplied the results of our cohort by 3.7 (the ratio of the number of B-notifications per year to the size of our hypothetical cohort). However, our model estimates the net present value of health benefits and costs over 20 years resulting from one year's expenditures. Over time, if the

same number of B-notifications entered the state, then (all else remaining the same) each year the costs of evaluation and treatment would be paid, but benefits would be realized from cohorts evaluated and treated in years past. We computed this by using the undiscounted simulation results.

2.5 Formal specification

We describe the structure of the continuous time, discrete event system which constitutes our model (using the terminology of Glasserman and Yao [7]). We specify four components: (1) the state space $\mathbf{X}$ of the model, (2) the set of active events for each state (i.e., the set of events which are possible when the system is in a given state), (3) the transition function which specifies how the state of the system changes when an event occurs, and (4) the parameter values and initial conditions. Each active event has an event time associated with it; the simulation proceeds by choosing the active event with the smallest event time. The state of the system is then transformed (updated) according to the specified transition function for the chosen event, and finally the simulation time t is updated to the the time of the event which just occurred. This process yields a sequence of event occurrence times $t^0, t^1, \ldots, t^k, \ldots$ and a corresponding sequence of state values at these times, i.e. $\mathbf{X}^0, \mathbf{X}^1, \ldots, \mathbf{X}^k, \ldots$ for each event time index $k = 1, 2, \ldots$. Thus, $\mathbf{X}^0$ is the initial condition of the system at time t^0; the first event occurs at time t^1 and causes the state of the system to change from $\mathbf{X}^0$ to $\mathbf{X}^1$, and so forth. When such a state change takes place, new events may become active (and their event times must be determined); other events may no longer be active (and they must be removed from the event list). We first specify the state $\mathbf{X}$ of the system, and then each of the possible events (and the transitions that result).

3 State Specification

3.1 States of individuals

We first specify the possible states of individuals in the model. Individuals are indexed by j, $j = 1, 2, \ldots, N(t)$, where $N(t)$ is the number of individuals in the simulation at time t. The state of individual j is represented by the j-th component of one of the five vectors given in Table 1. Age, diagnosis status, and death are straightforward; we discuss tuberculosis status and public health status in further detail below.

Public health status. We classify individuals into three categories of "public health status" as follows. We begin all simulations with $N(0) = 1000$ recently arrived immigrants eligible to be sought or evaluated for domestic B-notification follow-up. For individual j, $H_j(0) = 0$ if the person is part of this initial cohort; when these individuals (newly arrived immigrants with B-notification) are evaluated, sought for evaluation but not found, or fail to present for evaluation, the value of H_j becomes 1. Individuals for which $H_j = 1$ will not be sought for tuberculosis evaluation. Finally, individuals who are contacts of active cases enter the simulation with $H_j = 2$; when they are sought for evaluation (whether they are located or not), the value of H_j also becomes 1, since they will no longer be sought for evaluation.

Tuberculosis status. All individuals in the model are assumed to be in one of eight tuberculosis-related classifications (Figure 1): (0) individuals who are not at risk of development of active disease (barring re-exposure, which we neglect), (1) recently infected latently infected individuals at relatively high risk of progression to active disease, (2) individuals who are latently infected and at risk of progression, but who are not recently infected individuals at relatively high risk of progression, (3) individuals in ATS class 4 who are considered candidates for latent tuberculosis infection treatment (e.g. excluding former active cases who successfully completed

an adequate treatment regimen for their most recent episode of active disease), (4) smear-negative active disease, slowly progressing, (5) smear-positive active disease, slowly progressing, (6) smear-negative active disease, fast progressing, and finally (7) smear-positive active disease, fast progressing. For convenience, we also define C_j to be the "true" ATS class of the individual as indicated in the Table. The simplifying assumptions and parameter choices underlying this model structure will be discussed in more detail below.

Tuberculosis status of source of chain of infection. Whenever person j infects person j', the value of $O_{j'}(t)$ is set to $O_j(t)$. Thus, all individuals j who ever become active cases in a chain of infection resulting from individuals in ATS class 2 in the original cohort have $O_j = 2$, all individuals j whose chain of transmission began with an individual in ATS class 3 in the original cohort have $O_j = 3$, and all individuals j whose chain of transmission began with an individual in ATS class 4 in the original cohort have $O_j = 4$. Thus, prevention efforts which target class 4 (for example) could, in principle, eliminate all active cases for which $O_j = 4$.

Ethnodemographic grouping. We classified individuals into one of four ethnodemographic groupings: (1) African-American, (2) Asian-American, (3) Caucasian, or (4) Hispanic.

Source of infection. If person j infects person j', the value of $S_{j'}$ is set to j, $j = 1, \ldots, N(t)$. If person j is in the initial cohort, then $S_j = 0$. Observe that all the values of O_j can be determined from C_j and S_j.

3.2 Global variables

Additionally, for $i = 2, 3, 4$ corresponding to individuals with $O_j = 2$, $O_j = 3$, and $O_j = 4$, the following scalar variables are defined: L_i: the number of *lost* Quality-Adjusted Life-Years by individuals in the simulation (including both individuals in the original cohort, as well as contacts added to the cohort); F_i: the total cost; C_i: the cumulative number of diagnosed

cases; and M_i, the total number of deaths.

3.3 Full state space

The full state space of the system is specified by the collection

$$\mathbf{X} = \{B_j, H_j, A_j, U_j, D_j, S_j, \theta_j, L_i, F_i, C_i, M_i\},$$

where B_j is the collection of tuberculosis status values for each individual j, etc., and $i = 2, 3, 4$. We omit C_j and O_j, since they can be derived from the state space as given.

3.4 Initial conditions

The model is initialized with $N(0) = 1000$ individuals. We assume that all individuals are alive ($D_j = 0$ for all j), undiagnosed ($U_j = 1$ for all j). A fraction of individuals are assigned to each tuberculosis class, as discussed in the parameter values section; C_j is derived from B_j as shown in Table 1, and $O_j(0) = C_j(0)$. The age A_j is derived from the age distribution of the cohort (as given by the initial conditions), and the ethnodemographic distribution is given by that of the B-notification cohort.

We assumed a baseline cohort of 1000 individuals, assumed to be either (a) uninfected, (b) classified as TB2 according to the American Thoracic Society classification[8] (positive tuberculin skin test, but normal chest radiographic findings), (c) class TB4[8] (abnormal chest X-ray consistent with past tuberculosis, but no evidence of current active disease), (d) smear-negative or smear-positive active tuberculosis (class TB3). We constructed a hypothetical cohort informed by the following three sources of data: (1) a study of immigrants and refugees in San Francisco[9], (2) California B-notification reports, (3) unpublished program data from selected California tuberculosis control programs, and (4) CDC Information on Migrant Populations

(IMP) data. A study of San Francisco immigrants and refugees (1992–1993) found that approximately 6.9 percent of individuals with a B-notification were diagnosed as active cases, approximately 11.4 percent were in class TB2, 59 percent were in class TB4, and 18.7 percent had no evidence of infection (with three and one half percent unknown)[9]. Our uncertainty analysis range for the percent of active cases (class TB3) was three to six and nine-tenths percent, for the fraction of individuals in class TB4 was 54 to 69 percent, and for the fraction of individuals with latent TB infection was six to 16 percent. However, only 61 percent of the TB4s were reported to be eligible for further treatment (on the grounds that they had not had adequate prior treatment)[9] (we used an uncertainty analysis range of 56 to 66 percent). For all TB4s in the initial cohort, each variable G_j is set to 1 with probability given by the desired probability of treatment eligibility and to 0 otherwise; individuals becoming TB4s during the simulation due to self healing all have G_j set to 1. The California B-notification registry contains data for approximately 60 percent of individuals with B-notifications sent to California, although incomplete information included on such reports limits their generalizability at the present time.

4 Events

The full set of possible events is given in Table2. The event times and transitions associated with each of these will be discussed in turn. Any state variables (components of $\mathbf{X}$) whose values are not otherwise specified for any particular event are assumed to remain unchanged from $\mathbf{X}^k$.

4.1 Reactivation of LTBI

4.1.1 Risk of reactivation (progression)

For each individual j who is at risk of progression ($B_j = 1, 2, 3$), a progression event $\mathcal{P}_j$ is active.

For individuals in the fast progression category ($B_j = 1$) [2, 10], the tuberculosis progression time (the time at which the individual will develop tuberculosis, if no other event occurs first) is found by adding an exponentially distributed waiting time with mean $1/v_1$ to the current simulation time. We assumed that v_1 was 1 yr^{-1}; this value is somewhat arbitrary, as there are a number of plausible combinations of fast progression probability and the subsequent waiting time. Latently infected individuals at baseline who were not in this special fast progression category could either class TB2 or TB4 ($B_j = 2$ or $B_j = 3$); we modeled the annual risk of progression to disease in these individuals as declining exponentially with time[1]. The declining exponential form was chosen as a parsimonious way to model observed declines in incidence[11, 12, 13, 14]. The event time is then found by sampling the waiting time from a distribution characterized by exponentially declining hazard $\lambda(t) = \lambda_0 e^{-\alpha(t-\theta_j)}$ for $t > \theta_j$ (note that with probability $e^{-\lambda_0/\alpha}$, the waiting time is infinite and the event never occurs), and adding the waiting time to the current simulation time. The hazard is assumed to be λ_0 at time θ_j (the time of the last status change, i.e. when the individual entered the status $B_j = 2$ or $B_j = 3$).

For individuals in class TB2, the progression rate to disease was estimated from a study of untreated refugees arriving in Australia[12, 13, 14]; for this cohort, individuals with abnormal chest X-rays (and who may be considered putatively in class TB4) were excluded; the tuberculin skin test was administered with the Australian standard ten TU of PPD[15], and so the extent of induration is not directly comparable to tuberculin skin tests in the United States based on five

TU of PPD from different manufacturers[8]. We first determined the time change in the incidence rate by fitting a simple exponential curve to the overall incidence rate in the cohort[12]; we then scaled this curve by the incidence rate for individuals with 15 mm of induration or more (160.3 per 10^5[14]) and the reported overall incidence in the cohort. The incidence rate was modeled as $I_0 e^{-kt}$, where $I_0 = 217$ per 10^5 person-years and $k = 0.052$ (so that the hazard would become half its initial value in 13.3 years; for comparison, the authors reported that the incidence rate in the first three years for individuals with 15 mm or more of induration was 213 per 10^5 (95 percent CI 150–300)[12]). For sensitivity analysis, we assumed lower and upper bounds derived as follows. For the lower bound, we (1) used the lower overall rate reported for individuals with ten mm or more of induration (using ten TU of PPD)[14, 15], and (2) assumed that some of the incidence rate in the cohort was due to recent transmission. The incidence rate of tuberculosis due to recent transmission was 30 per 10^5 (from the incidence rate in individuals with less than ten mm of induration to ten TU)[12]. To determine the incidence in tuberculin-positive individuals that might be attributable to reinfection, we used a lower-bound estimate of 16 percent protection from disease due to reinfection[16] among the 58 percent of the cohort that were read as TST-negative[12]; this procedure yields an incidence rate I_0 of 142.3 per 10^5 with $k = 0.066$ (hazard becoming half its original value in ten and one half years). For the upper bound, we observed that the reported 95 percent upper confidence bound for the incidence rate in the first three years was 300 per 10^5; for simplicity, we chose the sensitivity analytic range as 217 ± 75 (142–292) per 10^5, and independently chose the half-time uniformly between ten and one half and 13.3 years; our "average scenario" is thus biased toward a more rapid falloff of risk than the overall cohort showed—a bias which reduces the calculated cost-effectiveness of the intervention. Finally, we observe that other studies of tuberculosis in latently infected individuals did not separate individuals with abnormal radiographic findings[17].

For individuals in class TB4, we began with data from the placebo arm of the IUAT trial of INH preventive therapy[11]. A fit of exponentially declining risk (chosen for parsimony) to these data would yield an annual risk of reactivation of approximately 430 per $100,000$, declining to half this value within three and six-tenths years ($I_0'e^{-k't}$, $I_0' = 4.3 \times 10^{-3}$ per person-year, $k' = 0.193$ per year). Such a rapid falloff rate of risk would, if extrapolated beyond the 5-year follow-up period of the IUAT trial to our 20 year analytic horizon, result in lower cumulative risk for individuals in class TB4[8] than in class TB2 over the duration of the study. To avoid assuming that individuals in class TB4 eventually have lower risk than individuals in class TB2, we assumed that when the declining hazard for a TB4 reached the initial level for an individual in class TB2, we used the hazard for TB2. Specifically, the hazard was assumed to be

$$h = \max\Big(I_0(\frac{I_0'}{I_0})^{\frac{k}{k'}}e^{-kt}, I_0'e^{-k't}\Big)$$

Other studies suggest that 600 per $100,000$ person-years reasonably estimates the progression rate in individuals with abnormal chest X-ray[18, 19]. Other studies[20] that reported higher values may not have removed active cases at the baseline of the study; for instance, a study conducted by US Health Departments[20] reported incidence rates of nearly three percent per year in the first year, declining to less than one percent per year (relative to the baseline number at risk) by the fifth year. Similarly, a study conducted in mental institutions[20] revealed an incidence rate of one and four-tenths percent during the first year, declining thereafter, but again cautioned that some of the high initial rate may have reflected a failure to exclude all prevalent cases at baseline; note also that ongoing transmission may have been higher in the earlier era. A study of reactivation tuberculosis in individuals previously treated[21] found low rates of reactivation, but the individuals had stable lesions for the preceding five years. For our uncertainty and sensitivity analysis, we chose a baseline initial annual risk of reactivation uniformly between 430

and 770 per $100,000$ (so that our average value[18, 19] is 600 per $100,000$, and the lower bound[11] is 430 per $100,000$); we chose the half-time (time it would take for the declining hazard to reach half its original value) uniformly from three and six-tenths years to twice this value. Finally, we note the likelihood that the initial cohort of refugees examined by Marks et al. contained not only latently infected individuals at lower risk, but some newly-infected latently infected individuals at higher risk. By assuming that there are no individuals in class $B_j = 1$ in our initial cohort, we assume the values derived by Marks et al. to all ATS class TB2 individuals in the initial cohort. For newly-infected individuals, we chose values of the probability of fast progression to TB and the rate of fast progression to give approximately 5% of active disease within two years (in the absence of intervention) [2, 10]. For convenience, we also applied the same progression rates given in Marks to all other ATS class 2 individuals not at high risk of progression, and observe that these rates are lower than those used in some other previous modeling studies [2] (making our results somewhat more conservative).

4.1.2 Development of disease

Individuals with disease in the model are classified as being sputum smear-positive or sputum smear-negative for acid-fast bacilli ("smear-positive" or "smear-negative," respectively). Smear status is important because it affects transmission, hospitalization, and mortality. Unfortunately, however, precise quantification of the natural history of smear progression does not appear to be available.

Data from repeated mass radiographic screening in Japan suggests that more than 50 percent of all newly-discovered smear-positive cases develop in less than one year[22], although it is unclear how many of these newly discovered active cases would have been due to new infection or to recent reinfection. Because of heterogeneity in the rate of development of smear positivity, and

the fact that some smear-negative individuals may nevertheless have serious disease, we followed previous models (e.g.[2]) in classifying individuals with pulmonary disease as progressing rapidly to smear-positivity or not progressing rapidly (or at all) to smear-positive status. Progression to smear-positivity before diagnosis is important because it represents a lost opportunity to have detected the case at a time when the individual was less infectious and potentially less likely to be hospitalized, as discussed earlier. We assumed that a fraction of individuals who develop tuberculosis will develop smear positive tuberculosis quickly (uncertainty analysis range 0.4–0.6); we denote the probability of being a "fast smear progressor" by ϕ.

Although individuals at baseline are assumed to have been screened for smear-positivity in the overseas examination, it is possible that some fraction of them are in the rapid progression category when they enter the country (either because they had already had active disease at the time of their overseas examination but had not become smear positive, or because they were infected or reinfected between the examination and their domestic follow-up); we assume, however, that all B-notification individuals are in the slow category. This is conservative (leading to potential underestimation of the benefits of screening), since we are assuming relatively little smear-positive disease may be averted by earlier detection of smear-negative cases.

Thus, whenever a progression event occurs, with probability ϕ the tuberculosis status $B_j = 4$ and with probability $1 - \phi$, $B_j = 6$.

4.2 Smear progression

An individual j with smear-negative disease may progress to smear-positive status (smear progression event $\mathcal{W}_j$. For individuals who will rapidly develop smear-positive disease, we assumed that the average time to develop smear-positive disease is two months (uncertainty analysis range: one month to one year); for individuals who will not rapidly develop

smear-positive disease, we assumed that the average time is more than one year (conditional on survival and not being treated; uncertainty analysis range: one to five years). When a smear progression event happens, if $B_j = 4$, then B_j is set to 5; if $B_j = 6$, then B_j is set to 7.

4.3 Self healing

Because of studies which suggested that 30 percent of individuals with active tuberculosis would undergo self-healing within five years[23] if left untreated, we modeled smear-reversion even in the absence of treatment. Although these rates are difficult to characterize by applying the results from the populations described in the literature to our population, if we had neglected the possibility that some (perhaps small number) of actively detected cases would have self-healed instead of becoming passively detected cases, we would have introduced some bias in favor of the health benefits and cost-savings of the domestic follow-up. We assumed a six percent self-cure rate for smear-negative individuals per year (waiting times are exponentially distributed). However, we assumed no self-cure nor smear-reversion for individuals with smear-positive disease. Finally, individuals who self-cured are assumed to become class TB4 and are assumed to have the same risk of reactivation; if self-healing occurs, then B_j is set to 3. Note that we assume that self-healed individuals are the same as individuals who begin the simulation in ATS class 4.

4.4 Death due to undiagnosed tuberculosis

Five-year survival rates for tuberculosis[24, 25, 26, 27, 23] during the pre-chemotherapy era ranged from approximately 0.19 to 0.49. These rates do not apply to the present time, since tuberculosis is a curable disease. Unfortunately, some patients are diagnosed only at death. We

separately simulated (1) deaths before diagnosis and (2) deaths of patients undergoing therapy (discussed under Passive diagnosis, below).

As shown in Table 5, a small fraction of individuals are diagnosed only at death. The values from Table 5 are biased estimates of the probability of dying from tuberculosis, since (1) some cases dead without a diagnosis may be missed, (2) some cases dying during tuberculosis may reflect other, background, causes of mortality unrelated to tuberculosis, and (3) some medical conditions, such as cancer treated with immunosuppressive drugs, may have led the individual to develop tuberculosis disease. Thus, the fraction dead at diagnosis may underestimate the probability of death before diagnosis (although even in this case, the underlying cause of mortality may not have been tuberculosis), and the fraction dying during therapy overestimates the fraction of deaths attributable to tuberculosis. We chose to assume death rates (force of mortality) of two to five percent per year, five through 20 percent per year, and 20 to 35 percent per year for untreated smear-negative tuberculosis for the three age groups (respectively); we assumed that the force of mortality due to active disease could be up to 50 percent higher in smear-positives without treatment.

Because the fraction of deaths in tuberculosis patients attributable to the disease has not been precisely estimated in the literature and cannot be estimated from available TB case reports, we estimated the annual risk of mortality for tuberculosis patients due to causes other than TB using California mortality figures, adjusted by sex and race. However, because some end-stage medical conditions may lead to the development of TB itself in previously infected individuals, we expect the death probability among TB patients from causes other than TB to be higher than for the general population. Several recent studies suggest that as many as one-third to one-half of deaths of older individuals with TB in low-morbidity settings may be related to other comorbid conditions (such as end-stage renal disease, heart disease, or cancer)[28, 29, 30], and we

consider this for our sensitivity analysis.

4.5 Natural history summary

In our model, undiagnosed tuberculosis patients are expected to be diagnosed passively with a waiting time of less than three months on average, as discussed in this Appendix. We modeled this as a competing exponential risk, and as the values we choose for the diagnosis rate are large compared to the mortality and self-healing rates, the dynamics are largely dominated by the waiting time to diagnosis. Consequently, precise characterization of the untreated natural history contributes little to the determination of the model outputs. For definiteness, however, we selected (arbitrarily) natural history parameters to yield (1) a 48 percent long run average proportion of active cases which were smear-positive, (2) a five-year case fatality rate of smear positive cases of approximately 50 percent, (3) a five-year case fatality rate of smear negative cases of approximately 45 percent, (4) approximately one third or less of active cases undergoing self-cure after five years [31]. It should be noted that these particular epidemiological summary parameters would be expected to differ considerably depending on the underlying demographic and socioeconomic conditions, especially the age structure and nutritional status of the population, and are used here simply as an approximate reference benchmark for untreated tuberculosis.

As discussed earlier, we assumed heterogeneity in smear-progression rates, choosing (1) a value of 12 per year (mean waiting time, one month) for the rate at which the fast smear-progressors move from smear-negative to smear-positive, (2) a value of 0.2 per year (mean waiting time, five years) for the rate at which the slow smear-progressors move from smear-negative to smear-positive, (3) a death rate of 0.333 per year for smear-positive active cases (survival time conditional on no self-healing, 3 years), (4) a death rate of 0.02 per year for (non-HIV-positive)

smear-negative individuals, (5) a self-healing rate of zero for fast smear-negative individuals, (6) a self-healing rate of 0.25 per year for slow smear-negative individuals, (7) a smear-reversion rate of 0.85 per year for fast progressing smear-positives, and finally (8) a smear-reversion rate of 2 per year for slowly progressing smear-positives. These particular parameter choices approximately reproduce the previously discussed epidemiological summary parameters.

4.6 Death due to unrelated causes

For any individual in the simulation, $\mathcal{M}_j$ is active provided only that the person is living. This event time was constructed using five-year ethnodemographic-class specific life tables, as follows.

Because recent improvements in reporting race/ethnicity have led to temporary inconsistencies in comparing California state demographic and disease reporting data, we chose to use year 1999 population and tuberculosis statistics for completeness and comparability (source: California Department of Finance, Demographic Research Unit, and California Department of Health Services, Tuberculosis Control Branch). Little is known about the age- and national-origin specific force of mortality due to causes other than tuberculosis in recent immigrants, and so we used the following simple method to estimate the mortality. First, we used California population estimates and reported deaths (subtracting tuberculosis mortality) to arrive at crude five-year survival probabilities for five-year classes from age 15 to age 85 (with 90 and above collapsed into one category) for African-American, Asian/Pacific Islander, Caucasian, and Hispanic categories. For this mortality adjustment, we assumed that the ethnic breakdown was the same as for all foreign-born tuberculosis cases; in 1999, this was two and twelve one-hundredths percent Black, 55.99 percent Asian/Pacific Islander, four and eleven one-hundredths percent Caucasian, and 37.78 percent Hispanic. Tuberculosis mortality is small

in California compared to all-cause mortality, however, and this adjustment is quite small.

For the B-notification cohort itself, we used country of origin as a proxy for the ethnic/demographic category (source: *CDC Information on Migrant Populations (IMP) data*; details available upon request), and thus assumed that 84 percent were Asian/Pacific Islanders, six percent were Caucasian, two percent were African-American, and eight percent were Hispanic. We used the same data to estimate the fraction under 15 years of age and in each five-year age category over 15. We assumed the age distribution was independent of ethnicity. For secondary infections, for simplicity we used the ethnic and age breakdown for all reported tuberculosis cases in California in 1999.

For each individual, we randomly chose a non-tuberculosis mortality time based on the ethnic composition of foreign-born tuberculosis cases, the age-distribution of the B-notification population, and the California population and mortality rates; when an individual is simulated to die of tuberculosis before this other-cause mortality time, the difference between the simulated TB death time and the other-cause mortality time is the number of years of life lost due to tuberculosis. This method is limited because (1) the age and national origin need not be independent, (2) foreign born tuberculosis patients may differ from B-notification arrivals, and (3) California ethnic population data need not apply to recent immigrants. For our uncertainty and sensitivity analysis, we assumed that the five-year death probabilities could vary within ten percent of their baseline values (i.e., all values could be multiplied by a factor between 0.9 and 1.1), and that moreover, the age distribution could vary by a range of two and one half years younger or older.

Table 4 gives approximate five-year survival probabilities for reference year 1999, using California Department of Finance population estimates (`http://www.dof.ca.gov/HTML/DEMOGRAP/Druhpar.htm`, accessed 22 September 2004);

because of the relatively small number of (secondary) cases in the Native American group, we excluded this group from the simulations. We assumed (based on CDC Information on Migrant Population data) that 83.9 percent of B-notifications were Asian-Pacific Islander, 8.3 percent Hispanic, 6.1 percent Caucasian non-Hispanic, and 1.7 percent African-American. Native Americans accounted for 0.44 percent of tuberculosis in 1999; these were excluded from the analysis and the remaining categories adjusted proportionally upward.

4.7 Transmission

New infection events from person j are active whenever person j is infectious and undiagnosed ($4 \leq B_j \leq 7$); we always assume that diagnosis is followed by therapy rendering the individual rapidly noninfectious. We assume that the waiting time to the next new infection event is exponential with a mean waiting time given by the reciprocal of the number of new infections per year for the given smear status.

Predictive inaccuracy in assessing the number of cases that may originate from each source case arises from three factors: (1) the unknown risk per unit time that may be experienced by a contact of an individual with tuberculosis (smear-positive or smear-negative), (2) the unknown number of contacts of each individual, and (3) the unknown latent tuberculosis infection status of each contact. We assumed that each smear-positive source case may infect between three and 13 new individuals per year[32], but that smear-negative cases infect individuals at a rate of anywhere from 0.05 to 0.3 times the rate assumed for smear-positive individuals[33, 34, 35]. Transmission is reduced by active case finding because patients spend less time in the community, and fewer of them will be expected to be or become smear-positive. A study of screening in the Netherlands[36] suggested that screening could reduce tuberculosis transmission

because passively detected patients and patients with a long duration since arrival were more likely to be first in an RFLP cluster than patients detected by screening or recent arrivals (but partially attributed the finding to confounding by duration of stay). A statistical analysis[37] of tuberculosis trends estimated the number of secondary cases caused by a tuberculosis case to be 0.55; assuming that 45 percent of cases were smear-positive over this period, and that smear-negative cases caused 0.22 times as much transmission as smear-positive cases implies that each smear-positive case causes approximately 0.963 new cases, and that each smear-negative case causes 0.21 secondary cases. This has the consequence that the total contact investigation costs are smaller for smear-negative cases[38]. In California, 2001, 0.9 percent of individuals found through contact investigations from smear-positive source cases had active tuberculosis; 0.5 percent of individuals found through contact investigations from smear-negative source cases were active cases[39].

Whenever a transmission event $\mathcal{T}_j$ occurs for person j at time t, N increases by 1. The individual is selected at random from the age and ethnic distribution of active cases; little is known about the age and ethnic distribution of new infections from individuals in a B-notification cohort. For newly infected individuals, $H_j = 2$.

4.8 Passive diagnosis

4.8.1 Time to diagnosis

Any individual with active tuberculosis ($4 \leq B_j \leq 7$) may be diagnosed passively; we assume an exponentially distributed waiting time. To model the diagnosis rates among passively diagnosed cases, we used several reports. No published study reports delays in diagnosis (time from symptoms to diagnosis) for recent immigrants to California. We used data reported from a

consecutive sample of active TB patients in Los Angeles[40]. The response rate in the survey was 60 percent; 72 percent of the patients in their survey were foreign born (foreign birth was not a significant predictor of delay). We assumed that smear-positive and smear-negative individuals were diagnosed with a mean waiting time to diagnosis of 74 days (uncertainty analytic range: 64–84 days); this may overestimate the delay for smear-positive persons, since at least one study has found less delay for smear-positive persons than for smear-negative[41].

Whenever an individual is passively diagnosed, the individual is treated and (we assume) rendered noninfectious. The individual may be hospitalized, and may die during therapy. For simplicity, all events that follow diagnosis are assumed to happen at the time of diagnosis, at which time we account for (1) lost QALYs, (2) costs, (3) the diagnosis itself, and (4) the mortality of an individual with tuberculosis. For the simulation, U_j is set to zero, and then the variables L_{O_j}, F_{O_j}, C_{O_j}, and M_{O_j} are incremented by determining the number of future discounted lost QALYs and costs (based on the smear-status and death rates during therapy for individuals based on age and smear status), multiplying these quantities by $e^{-\delta t}$ (where δ is the discount rate and t the current simulation time), and adding these to L_{O_j} and F_{O_j} respectively. The quantity C_{O_j} is incremented by $1 \times e^{-\delta t}$ at the time of diagnosis, and the quantity M_{O_j} is incremented by the probability of death (derived from the decision tree) times $e^{-\delta t}$.

Specifically, each decision tree in our model corresponds to a sequence of random multinomial choices between alternatives with probabilities as indicated. A vector of outcome variables is given at each terminal node, indicating what costs, lost QALYs, etc. are accumulated should the individual's choices end at that particular terminal node.

For passively diagnosed cases, we compute the number of lost QALYs, the expected cost, and the probability of death. (With different parameters, this decision tree will be used to compute these three quantities for actively diagnosed cases, i.e. those found through domestic

B-notification follow-up or contact investigation.) The probabilities of hospitalization are given in Table 7.

4.8.2 Death during treatment

California tuberculosis case reports provide an estimate of the fraction of cases diagnosed at death, and of the fraction of individuals with TB who die while having the disease. The fraction of TB cases not known to be AIDS cases in the years 1996 through 2000 who die during therapy or who are dead at diagnosis is given in Table 5; these fractions are based on final California outcomes. Because the vast majority of cases in California are passively detected, we chose these overall death rates to be the death rates for passively detected cases, varying for uncertainty analysis by plus or minus 20 percent of the baseline value. Actively detected cases were assumed to have the same mortality rates as passively detected cases for the baseline scenario.

4.8.3 Hospitalization

Some of the benefits of screening programs stem from the earlier detection of active cases, as has been assumed in other cost-effectiveness analyses[38]. A Netherlands study[42] found that 60 percent of passively detected cases were hospitalized, as opposed to 20 percent of actively detected cases; because the subjects were not randomly allocated to screening, some of the differences may however be attributable to selection bias. Using data from this study[43], we assume that the probabilities of hospitalization (with 95 percent confidence intervals) are proportional to the following: actively detected, smear-negative cases: 0.081 (0.036, 0.15); actively detected, smear-positive cases: 0.35 (0.23, 0.48); passively detected, smear-negative cases: 0.51 (0.36, 0.66); and passively detected, smear-positive cases: 0.66 (0.52, 0.78). We

used these values (± 10 percent) in the uncertainty and sensitivity analysis, subject to the constraint that smear-positive individuals are more likely to be hospitalized than smear-negative individuals (of the same mode of case detection), and that actively detected cases are less likely to be hospitalized than passively found cases (if the smear status is the same).

4.8.4 Costs of active TB disease

Outpatient tuberculosis treatment costs for pansensitive (non-drug resistant) tuberculosis were determined from Medicare physician fee schedules for California geographic pricing regions (averaged by the 2004 tuberculosis case count in each region), average nationwide Medicare Part B allowed charges, or in some cases, Medi-Cal reimbursements or the literature, as summarized in Table 1 of the main text. This includes the cost of drugs as well as the costs of directly observed therapy, medical follow-up, and laboratory work.

To estimate the costs of hospitalization for tuberculosis, we used data from the CDC Costs of Tuberculosis Hospitalization study[44, 45]. Because the costs of multidrug-resistant tuberculosis are large and may vary considerably from individual to individual, we have conservatively chosen to ignore any potential cost-savings that may result from earlier detection of INH-monoresistant and multidrug-resistant tuberculosis. Finally, in an older study of the costs of tuberculosis[46], the mean length of stay was found to be 19.9 days; assuming our value of $1,465 per day (2004 dollars) yields costs somewhat larger than the more recent CDC results we used[44].

To estimate the costs of sputum collection and analysis, we assumed that specimen collection, specimen concentration, AFB smear, and mycobacterial culture were undertaken every time. However, we assumed that mycobacterial identification and mycobacterial susceptibility testing could only be performed on culture-positive specimens, and we assumed that 63.5% of such specimens were culture-positive [47].

Where possible, 2004 Medicare Part B charges were used for California (computing a weighted average over the nine Medicare geographic pricing regions in California based on the number of cases of tuberculosis seen in that region in 2004). Where Medicare Part B charges were not available for 2004 (such as DOT and nurse refill labor), we used current Medi-Cal reimbursement rates (source: California Department of Health Services). Little information is available to determine the cost of nurse symptom review or prescription refill visits; current Medi-Cal reimbursement rates are approximately in agreement with one-half hour of staff RN time (using a recent salary survey [48]). Our use of Medi-Cal reimbursement rates to estimate DOT costs may be conservative, underestimating the true cost of a visit. We assumed a fixed fraction of patients on DOT, and did not need to adjust the expected costs of tuberculosis for the cost-saving features of the use of DOT [50, 51]. Moreover, the average cost of DOT may depend strongly on the assumed mix of in-clinic DOT and DOT outside the clinic [49]; we have made no effort to adjust for this.

Because tuberculosis prevention can yield cost-savings, we chose, in general, a very conservative cost accounting for active disease to avoid overestimating these cost savings; we do not consider the extension of therapy beyond six months, or case management costs that extend beyond the medical visits we assumed.

4.9 Utilities

Ongoing studies have demonstrated the feasibility of conducting quality of life measurements among tuberculosis patients and individuals undergoing therapy for latent tuberculosis infection [52, 53]; currently available literature, however, does not precisely quantify these health state utilities. We reviewed existing literature and chose convenient values based on a consensus of

the existing literature, supplemented with sensitivity analysis. Despite the importance of using quality adjustments to combine hepatitis and tuberculosis outcomes into a single health state measure, our unavoidable use of imprecisely measured quantities remains a limitation of our analysis.

Tuberculosis in the hospital, fatal cases. In previous analyses, researchers assumed a difference between severe or fatal tuberculosis, and less severe or non-fatal tuberculosis. Previous values for fatal or severe tuberculosis ranged from 0.21 [54] to 0.5 [1]. We assume that hospitalization time is 20 days; we chose the lower of these two values for the utility of ultimately fatal tuberculosis. Based on the value of 0.21, we estimate 0.043 lost QALYs for this period of hospitalization, a value far smaller than the discounted future life-years lost in fatal cases.

Tuberculosis in the hospital, nonfatal cases. Non-fatal tuberculosis health state utilities in previous articles has ranged from 0.66 [54] to 0.85 [1]. Hospital confinement for tuberculosis was determined (by use of a time-tradeoff questionnaire among members of the general population) to have a utility of 0.6 [55]; we chose this value because it was derived from a population survey. Inpatient tuberculosis (without mention of severity) was assumed to have a utility of 0.87 in another report [56]. We assume that hospitalization time is 20 days. Based on the figure of 0.6, we compute that 0.021 QALYs are lost for hospitalization of nonfatal tuberculosis cases.

Tuberculosis under outpatient treatment. One report estimated the utility of outpatient tuberculosis to be 0.89 [56]; another assumed that outpatient therapy had a utility of 0.9 [57]. A recent feasibility/reliability study of health state utility measurement suggests a median utility of 0.925 among tuberculosis patients on an initial interview, excluding those with comorbid conditions [52, 53]. We chose the median of these, 0.9.

Symptomatic tuberculosis prior to diagnosis. For undiagnosed tuberculosis, we simply used the disutility from a previous study [58] in the absence of precise quantification in the literature; this value, 0.9, was the same that we chose for tuberculosis under outpatient treatment.

Isoniazid side-effects, other than hepatitis. One previous study assumed that everyone who takes INH loses 0.01 from their health state utility [1], while a physician proxy survey found that individuals undergoing therapy for TB infection had a health state utility of 0.93 [54]. We chose to assume that individuals with side-effects due to isoniazid sufficient to warrant discontinuation lost 0.1 from their health state utility [58], and that there was no disutility from pill-taking alone in the absence of side-effects. For sensitivity analysis, however, we assumed that other side-effects could lead to 0.01–0.1 in lost utility, and that pill taking alone could lead to 0–0.01 in lost utility. We also assume that the duration of side-effects that warranted discontinuation of therapy was two weeks.

Isoniazid-induced hepatitis, hospitalization. For fatal hepatitis, we again followed the estimate from a physician proxy survey [54] of 0.12. For non-fatal hepatitis, this same survey yielded the value 0.62, while a different study utilized the value 0.85 [1]; we chose the median of these utilities, or 0.735, and assumed a duration of one month on average [1].

Parameter summary

For simplicity, we assume that outpatient cases in therapy lose 0.05 QALYs (based on six months of therapy at a utility of 0.9). For non-fatal cases of tuberculosis that are hospitalized, we assume 20*0.4 healthy days lost due to the hospitalization (0.021 QALYs, varied over the range 0.0105 to 0.042), and for fatal cases, we assume that 0.043 QALYs are lost (varied from

0.0215 to 0.086). We assumed that isoniazid side-effects other than hepatitis could result in a disutility (one minus the health state utility) of 0.01, but ranged this from 0.01 to 0.1. We assumed that there was no disutility for pill taking alone, but varied this from 0 to 0.01. We assumed that the utility of outpatient hepatitis was 0.62–0.85, with the base case of 0.735 (the midpoint). Finally, we assumed that the utility for hospitalized hepatitis was 0.6, but used a range of 0.21 to 0.735 for sensitivity analysis.

4.10 Domestic B-notification evaluation

Beginning with the arrival of a new immigrant with tuberculosis B-notification in the United States, the individual may or may not be evaluated, may or may not be an active case or have latent tuberculosis infection, may or may not be identified as having LTBI, may or may not start therapy, and may or may not complete therapy. We compute the expected lost QALYs, the expected cost, the number of cases of tuberculosis, the expected number of deaths (including those from adverse reactions to LTBI preventive therapy with INH), and the probability of starting and completing therapy, using the decision trees in Figures 2–5.

Figure 2 represents the process of evaluation (including the option of no effort, but also letters, phone calls, and home visits). Failure to evaluate an individual is indicated by a labeled terminal node; evaluation of an individual is represented by the gray circle containing the numeral "1"; the decision tree is continued in Figure 3 (beginning with the numeral "1" on the left.) We assumed that the individual would either present for evaluation with no effort on the part of the public health authorities, or that some effort would be needed (discussed below), which may or may not have been successful. Only a fraction, in general, are actually evaluated in reality, and we model this by choosing a certain fraction to be evaluated (the remainder not being

evaluated), and removing all individuals from the pool of new arrivals with B-notification. Mathematically, we assume that whenever the event $\mathcal{B}_j$ occurs, the individual moves from $H_j = 0$ to $H_j = 1$.

Follow-up costs for the initial evaluation were derived from a cost study[59] in B-notification in Santa Clara County, 1995–6. Out of 323 B-notifications in Santa Clara County in the study period, 79 were screened without interventions, 213 responded to letters, 17 to phone calls, and five to home visits (for a total of 314). We used these frequencies and costs to assess the costs of the initial evaluation. (Because the cost of mailing letters, conducting phone calls, and conducting home visits were not medical costs, we inflated these according to the all-items US Consumer Price Index.) We also arbitrarily assumed an administrative cost of $10 to open a file on each individual whether or not the individual is found and evaluated.

Figure 3 provides a decision tree for the domestic evaluation intervention we modeled, which includes radiographic evaluation and the tuberculin skin test (TST), as well as symptom review and laboratory work as needed. Note that Figure 3 includes the possibility that infected individuals may be misclassified as uninfected (TB0), as well as the possibility that uninfected individuals may be misclassified as infected (TB2) and given isoniazid prophylaxis.

We chose the probability that an active case found through B-notification would be smear-positive uniformly from the range zero to 15 percent (with a base case value of 7.5 percent). This conservatism partially accounts for the possibility that improvements in the program which result in shorter times between the overseas screening examination and the domestic follow-up may reduce the fraction of smear-positive individuals. For comparison, the yield of active case-finding was estimated by an older Netherlands-based trial of active case-finding in (primarily non-immigrant) persons with inactive tuberculosis or fibrotic lesions[60]; this population of individuals at risk was older than the California B-notification population, and

contains many individuals being followed up after treatment for tuberculosis disease. In the older Netherlands trial, individuals were allocated to either a control group or to a group examined every year; 28 cases were detected by active case-finding (two smear-positive), and twelve (two smear-positive) in the passively-followed group (this difference in smear-positivity was not statistically significant; the total number of cases was, however, small).

In the absence of a gold standard for the detection of LTBI, several estimates for the sensitivity and specificity of the tuberculin skin test have been derived. Conservatively, we assumed a sensitivity of 93 percent (with a cutoff of ten mm of induration) in the general population[61], but for sensitivity analysis, assumed a sensitivity of 95% [62]. We also assume that the tuberculin skin test (TST) with a cutoff of ten mm of induration has a specificity of 99 percent in the general population[61, 63] to detect latent tuberculous infection (LTBI), assuming the absence of BCG vaccination. The leading countries of origin for individuals with a California B-notification are the Philippines, Vietnam, and China[47], all nations which report high levels of BCG vaccination (`http://www.who.int`). Because vaccination with BCG may cause false-positive tuberculin skin tests[64], for sensitivity analysis, we assumed that 50 percent of new immigrants would have received BCG vaccination, and that 25 percent would have ten mm or more of induration[38] even if uninfected. Thus, the overall specificity [38] would be approximately 0.87. We do not assume increased sensitivity to detect tuberculosis infection in individuals who have received BCG. We apply these values for the sensitivity and specificity of the TST to individuals with normal chest X-rays; we assume that chest X-ray screening identifies all individuals with abnormal chest X-rays consistent with active or inactive tuberculosis (class TB4). Figure 4 provides a decision tree for starting therapy and completing therapy, including the provision of directly observed preventive therapy (DOPT). As discussed in the text, in some scenarios we consider the possibility that a small fraction of the patient population is at high risk for noncompletion of

their therapy (and are thus considered candidates for targeted DOPT).

Individuals who are diagnosed with LTBI decision tree Figure 4, and then proceed to Figure 5 if they start therapy. The parameters (in particular the probability of completion of therapy) in Figure 5 depend on whether the patient was a candidate for DOPT and whether DOPT was provided. Figure 5 begins with isoniazid prophylaxis, and also includes the possibility that an individual on isoniazid prophylaxis will develop hepatitis or will experience no hepatitis; we assume individuals with hepatitis will either be hospitalized or not, and will live or die, but will not complete therapy and will receive no benefit from it. Individuals without hepatitis experience "minor or no adverse" events (which do not affect their probability of completion, or experience "adverse" outcomes or events which do affect their probability of completion. Finally individuals may either complete therapy or not; we assume that individuals who complete therapy are either cured or not. (In practice, it cannot be determined who is cured or not.)

4.10.1 Rates of starting therapy

We assume a range of between 55 percent and 85 percent of eligible patients who start therapy, based on unpublished reports from California health jurisdictions serving a wide variety of populations. Unfortunately, less is known about the cost and efficacy of interventions to increase the fraction of individuals who start therapy. For sensitivity analysis, we chose to assume that a $10,000 investment in evidence-based education to health care providers who see patients with latent tuberculous infection could improve starting rates by ten percent relative to baseline (see the British Evidence Based Outreach trial[65]).

4.10.2 Rates of completion of therapy

Rates of completion of therapy in general depend on the population served as well as the level of adverse effects of isoniazid therapy. We assumed that individuals without adverse effects would complete therapy at a rate of anywhere from 45 percent to 80 percent, based on unpublished reports from selected California health jurisdictions. As indicated in the text, we either assumed that (1) the cost of improved adherence is computed from the cost of the additional medications and nurse refill visits, and so forth, or that (2) the cost of improved adherence includes not only medications, nurse refill visits, but also additional costs needed to maintain adherence for patients who otherwise would not have completed their therapy. Specifically, we assumed that a small fraction of the population (ten to 20 percent) would require Directly Observed Preventive Therapy (DOPT). We assumed that individuals on DOPT were twice as likely to complete their therapy[66] as they would have been without DOPT; the cost of DOPT was assumed to be the same as that of DOT for tuberculosis disease (since Medi-Cal reimbursement rates are the same).

4.10.3 Efficacy of LTBI therapy

The IUAT isoniazid preventive therapy trial[11], a randomized double-blind placebo-controlled trial of INH chemotherapy for latent tuberculosis infection in older individuals with small, stable radiographic lesions, provided estimates for the efficacy of INH therapy of latent tuberculosis infection. The group completing twelve weeks of therapy had the incidence rate reduced by 21 percent, 24 weeks by 65 percent, and 52 weeks by 75 percent. (Note that for those who completed their regimen and were compliant, the efficacies for twelve weeks, 24 weeks, and 52 weeks were 31 percent, 69 percent, and 93 percent, respectively.) For individuals completing

three months of INH, we assumed an efficacy of 21 percent, for six months, 65 percent, and for nine months, 70 percent (using the midpoint of the 24- and 52-week values). We assumed that some fraction of individuals who did not complete the full regimen completed enough therapy to receive benefit; for definiteness, we assumed that 30% of individuals who did not complete the full regimen completed four months of therapy and we conservatively assumed that the efficacy of this partial regimen was 21%.

4.10.4 INH resistance

We assumed that individuals with INH-resistant latent tuberculosis infection (whether with added rifampin resistance or not) would not benefit from INH chemotherapy, and that the probability of INH-resistant latent tuberculosis infection was independent of other factors influencing the success of chemotherapy. We assumed that the probability of (any) INH-resistant latent tuberculosis infection was 12.9 percent (using surveillance reports for recent immigrants from China, Vietnam, and the Philippines, 2002). This includes multidrug-resistant strains. Drug-resistance is important in our model primarily because individuals with INH-resistant latent tuberculosis infection are assumed not to benefit from chemotherapy; we have conservatively chosen to ignore differences in mortality rates and treatment duration for individuals who have drug-resistant tuberculosis.

4.10.5 Adverse effects of INH therapy

Adverse effects, including hepatitis, may result from the use of isoniazid (INH) in therapy for latent tuberculosis infection. The probability of INH-related hepatitis is not precisely known, and depends on the age of the patient as well as on the criteria for defining hepatitis[67]. Evidence suggests that liver damage is more severe if INH is continued after the onset of symptoms,

e.g.[68], and thus monitoring of patients for symptoms is routinely undertaken. For those starting INH chemotherapy, we used data from a prospective cohort study to assess the age-group specific fraction who develop INH-associated hepatitis[67], using a definition which included the occurrence of symptoms which resolved when INH was discontinued (and not laboratory findings only). These probabilities (with 95 percent exact confidence bounds given in parentheses[69]) were approximately 0.8 (0.3–1.8) per thousand for ages 15–34, 2.1 (0.6–5.5) per thousand for ages 35–64, and 2.8 (0.07–15.4) per thousand for age 65 and older; for uncertainty/sensitivity analysis, we sampled each of these probabilities from the beta distribution reflecting the posterior density based on the Nolan findings[67] assuming a binomial outcome and uniform prior density. Studies based on laboratory tests (e.g.[70, 71, 72] give higher hepatitis rates, but the significance of asymptomatic hepatitis cases discovered by laboratory methods only is unclear. The IUAT trial itself reported 0.5 percent of individuals beginning isoniazid chemotherapy for latent tuberculosis infection developed hepatitis[11]; monitoring was however not consistent. A public health clinic based study of isoniazid preventive therapy, in which cases were identified by symptom monitoring together with laboratory tests for patients for whom the laboratory tests were recommended, found an overall hepatitis rate of 0.003 (0.3 percent) [73].

4.10.6 Hospitalization and death due to hepatitis

The probability that an INH-induced hepatitis case will require hospitalization (seven days[74, 75]; sensitivity analysis range seven to 21 days) is assumed to be approximately ten percent[74, 67]. The age-specific case-fatality rate of INH-induced hepatitis is not known; we assumed that the probability of death for hospitalized individuals of any age was 12.5 percent. The overall probability of death for individuals under age 35 who start therapy, using the parameters in Table 7 is then 10^{-5}, the same as used for all individuals starting therapy used in other

analyses[75]; the corresponding case-fatality rate we used is 1.25 percent, less than the 3.1 percent used as the base case in an earlier study[74]. Using the same case-fatality rate in all age groups ensures that older individuals then have a higher probability of death upon starting treatment because the probability of hepatitis is greater. Precise quantitative estimates of the duration of hepatitis symptoms under conditions of careful monitoring (and discontinuation of INH at the beginning of adverse events) are not available; conservatively, we chose to assume two weeks of relatively mild symptoms and two weeks of severe symptoms at the beginning (as suggested by case reports from unmonitored patients who continued to take INH after the onset of hepatic symptoms[68]).

4.10.7 Other adverse side-effects

Finally, we assumed a further probability of seven percent of having other adverse side-effects severe enough to warrant discontinuation of therapy [73]. We assumed these effects would last, on average, two weeks, and that a medical visit would occur as a result.

4.10.8 Cost of INH therapy for LTBI

We used Medi-Cal (California Medicaid) reimbursement data to estimate the costs of screening and treatment of latent tuberculosis infection. Costs are summarized in the main text. All dollars were adjusted to 2004 dollars using the Medical component of the Consumer Price Index. The duration of therapy in individuals who discontinued therapy early was assumed to be two months (based on unpublished data from selected California jurisdictions). Currently, a 9-month regimen of daily isoniazid is recommended for latent tuberculosis infection[76], and therefore we assume that individuals who completed therapy received nine months of therapy.

The cost of INH for latent tuberculosis infection was determined from the Medicaid Federal

Upper Limit price of $8.90 per 100 tablets (`http://www.cms.hhs.gov/medicaid`). The cost of drugs used to treat active tuberculosis was assumed to be $1118.33 (in 2004 dollars), based on the standard four-drug regimen (isoniazid, rifampin, pyrazinamide, and ethambutol for two months, followed by isoniazid and rifampin for four further months) for a 65-kg adult (using Red Book wholesale prices for 2004). We assumed that one doctor visit would be required for important isoniazid side-effects other than hepatitis, and that three doctor visits would be required for hepatitis. We also assumed that in the event of isoniazid-induced hepatitis, three liver function panels would be required.

4.11 Contact investigation

Contact investigation costs result from the initial disease control investigation, as well as the cost of identifying and treating latent tuberculous infection and disease among contacts. Our model structure yields lowered contact investigation costs for smear-negative individuals, since fewer of their contacts will have evidence of latent infection or disease[38]. To estimate the costs of the disease control investigation, we assumed twenty hours of time of a Licensed Practical Nurse (LPN) [51]. The results of an annual Nursing Salary Survey [48] suggest an average LPN salary (in 2004) of $\$32\,200$; assuming a $2\,080$ hour year and 25% of the base salary in benefits yields a total cost of $\$387.02$. Brown et al.[46] also estimated the mean cost per contact traced to be $17, which would yield smaller costs than we assumed (assuming ten contacts). We arbitrarily assumed that 80 percent of contacts would be found through investigation.

Selected natural history parameters are given in Table 6.

5 Uncertainty and Sensitivity Analysis

Using the parameter values in Tables 6 and 7, together with the costs in the main text, we chose a Latin Hypercube sample of size 1000 [77, 78, 79] from the parameter space and replicated the simulation 1000 times for each parameter set. From each collection of 1000 replications at each parameter set, we computed the number of QALYs saved, the net cost, the number of cases averted, and the number of deaths averted (discounted at 3%). These results are summarized in Table 8. Observe that there is considerable uncertainty in the net costs for the active case finding component resulting from the choice of parameter values.

To understand which parameters contribute to the uncertainty, we computed the partial rank correlation coefficient (PRCC) for each parameter and each outcome variable of interest, holding the other parameters constant [77, 78, 79]. Large absolute values of this correlation coefficient (near 1 or -1) indicate a strong dependence of the parameter on the outcome of interest. Briefly, we found that the most important parameter contributing to uncertainty in the number of QALYs saved by active case finding was the number of cases to be found, specifically, the fraction of active cases (which we varied from 0 to 0.07); the PRCC was -0.96. Similarly, the fraction of active cases was also the most important parameter contributing to the uncertainty in the net cost for active case finding, and the PRCC was -0.98. We also found that the poorly characterized natural history parameter for the rate of developing smear-positive disease from smear-negative cases contributed considerably to uncertainty in the number of QALYs saved by active case-finding (PRCC: -0.74), as did the mortality ratio between actively found and passively found cases (PRCC: 0.52). Other important parameters for the net cost for active case finding were the hospitalization cost for TB (PRCC: -0.55) and the costs not related to hospitalization (PRCC: 0.92). For the total number of cases prevented by active case finding,

we found that the most important parameters were the initial active case fraction (PRCC: -0.73), the transmission rate (PRCC: -0.58), and the probability of developing TB quickly (PRCC: -0.51).

We conducted an additional sensitivity analysis in which we varied the number of lost QALYs due to INH pill taking (even in the absence of side-effects severe enough to warrant discontinuation). We assumed that this went from 0 to a very high value of 0.01 (a disutility of 0.12 applied for one month). Over this extreme range, the partial rank correlation for the change in the number of QALYs due to treatment of TB4s was 0.98, and for TB2s was 0.94.

In Figure 6, we plot the net costs and the number of QALYs saved for each scenario. Scenarios for which the active case fraction was greater than 6% are symbolized by a solid triangle, and these almost all result in net cost savings, and many correspond to a larger number of QALYs saved. Scenarios for which the active case fraction was less than 1% are symbolized by a solid circle, and all such scenarios correspond to a positive net cost and frequently a low number of QALYs saved as well. The overall graph shows an inverse relationship, despite the fact that for each scenario, increasing expenditure never causes a net expected loss in QALYs (for this parameter range).

References

1. Tsevat J., Taylor W. C., Wong J. B., Pauker S. G.. Isoniazid for the tuberculin reactor: take it or leave it *American Review of Respiratory Disease.* 1988;137:215–220.

2. Blower S. M., McLean A. R., Porco T. C., et al. The intrinsic transmission dynamics of tuberculosis epidemics *Nature, Medicine.* 1995;1:815–821.

3. Porco T. C., Blower S. M.. Quantifying the intrinsic transmission dynamics of tuberculosis *Theoretical Population Biology.* 1998;54:117–132.

4. Murray C. J. L., Salomon J. A.. Modeling the impact of global tuberculosis control strategies *Proceedings of the National Academy of Sciences, USA.* 1998;95:13881–13886.

5. Weinstein M. C., Zeckhauser R.. Critical ratios and efficient allocation *Journal of Public Economics.* 1973;2:147–157.

6. Weinstein M. C., Stason W. B.. Foundations of cost-effectiveness analysis for health and medical practices *New England Journal of Medicine.* 1977;296:716–721.

7. Glasserman P., Yao D. D.. *Monotone structure in discrete-event systems.* New York: J. Wiley 1994.

8. American Thoracic Society . Diagnostic standards and classification of tuberculosis in adults and children *American Journal of Respiratory and Critical Care Medicine.* 2000;161:1376–1395.

9. DeRiemer K., Chin D. P., Schecter G. F., Reingold A. L.. Tuberculosis among immigrants and refugees *Archives of Internal Medicine.* 1998;158:753–760.

10. Porco T. C., Small P. M., Blower S. M.. Amplification dynamics: prediction the effect of HIV on tuberculosis outbreaks *Journal of Acquired Immune Deficiency Syndromes.* 2001;28:437–444.

11. International Union Against Tuberculosis Committee on Prophylaxis . Efficacy of various durations of isoniazid preventive therapy for tuberculosis: five years of follow-up in the IUAT trial *Bulletin of the World Health Organization.* 1982;60:555–564.

12. Marks G. B., Bai J., Simpson S. E., Sullivan E. A., Stewart G. J.. Incidence of tuberculosis among a cohort of tuberculin-positive refugees in Australia *American Journal of Respiratory and Critical Care Medicine.* 2000;162:1851–1854.

13. Marks G. B., Bai J., Simpson S. E., Stewart G. J., Sullivan E. A.. The incidence of tuberculosis in a cohort of South-East Asian refugees arriving in Australia 1984–94 *Respirology.* 2001;6:71–74.

14. Marks G. B., Bai J., Stewart G. J., Simpson S. E., Sullivan E. A.. Effectiveness of postmigration screening in controlling tuberculosis among refugees: a historical cohort study, 1984–1998 *American Journal of Public Health.* 2001;91:1797–1799.

15. Marks G. B.. Personal communication 2004.

16. Vynnycky E., Fine P. E. M.. The natural history of tuberculosis: the implications of age-dependent risks of disease and the role of reinfection *Epidemiology and Infection.* 1997;119:183–201.

17. Comstock G. W., Woolpert S. F., Livesay V. T.. Tuberculosis studies in Muscogee County, Georgia. Twenty-year evaluation of a community trial of BCG vaccination *Public Health Reports.* 1976;91:276–280.

18. Daňková D.. Tuberculosis risk in persons with fibrotic x-ray lesions *Bulletin of the International Union against Tuberculosis.* 1972;47:145–150.

19. Steinbrück P.. Tuberculosis risk in persons with fibrotic x-ray lesions *Bulletin of the International Union against Tuberculosis.* 1972;47:135–144.

20. Edwards L. B., Doster B., Livesay V. T., Ferebee S. H.. Tuberculosis risk in persons with fibrotic x-ray lesions *Bulletin of the International Union against Tuberculosis.* 1972;47:151–156.

21. Falk A., Fuchs G. F.. Prophylaxis with isoniazid in inactive tuberculosis. A Veterans Administration cooperative study XII *Chest.* 1978;73:44-48.

22. Toman K.. *Tuberculosis case-finding and chemotherapy. Questions and answers.* Geneva: World Health Organization 1979.

23. Grzybowski S., Enarson D. A.. The fate of cases of pulmonary tuberculosis under various treatment programmes *Bulletin of the International Union against Tuberculosis.* 1978;53:70–75.

24. Sampson H. L.. Fatality rates in pulmonary tuberculosis. Their trend based on roentgenological studies of 8,000 patients *American Review of Tuberculosis.* 1939;40:71–84.

25. Berg G.. The prognosis of open pulmonary tuberculosis *Acta tuberculosea Scandinavica, Supplementum.* 1939;IV:1–207.

26. Thompson B. C.. Survival rates in pulmonary tuberculosis *British Medical Journal.* 1943;2:721.

27. Tattersall W. H.. The survival of sputum-positive consumptives. A study of 1,192 cases in a county borough between 1914 and 1940 *Tubercle.* 1947;28:85–96.

28. Walpola H. C., Siskind V., Patel A. M., Konstantinos A., Derhy P.. Tuberculosis-related deaths in Queensland, Australia, 1989–1998: characteristics and risk-factors *International Journal of Tuberculosis and Lung Disease.* 2003;7:742–750.

29. Xie H. J., Enarson D. A., Chao C. W., Allen E. A., Grzybowski S.. Deaths in tuberculosis patients in British Columbia, 1980–1984 *Tubercle and Lung Disease.* 1992;72:77–82.

30. Fielder J. F., Chaulk C. P., Dalvi M., Gachuhi R., Comstock G. W., Sterling T. R.. A high tuberculosis case-fatality rate in a setting of effective tuberculosis control: implications for acceptable treatment success rates *International Journal of Tuberculosis and Lung Disease.* 2002;6:1114–1117.

31. Olakowski T.. Assignment report on a tuberculosis longitudinal survey, National Tuberculosis Institute, Bangalore, WHO Project: India 0103. SEA/TB/129 tech. rep.World Health OrganizationGeneva 1973.

32. Stýblo K.. Tuberculosis control and surveillance in *Recent Advances in respiratory medicine, Number 4* (Flenley D. C., Petty T. L.. , eds.):77–108Edinburgh: Churchill Livingstone 1986.

33. Grzybowski S., Barnett G. D., Styblo K.. Contacts of cases of active pulmonary tuberculosis *Bulletin of the International Union against Tuberculosis.* 1975;50:90–106.

34. Behr M. A., Warren S. A., Salamon H., et al. Transmission of *Mycobacterium tuberculosis* from patients smear negative for acid-fast bacilli *Lancet.* 1999;353:444–449.

35. Hernández-Garduño E., Cook V., Kunimoto D., Elwood R. K., Black W. A., FitzGerald J. M.. Transmission of tuberculosis from smear negative patients: a molecular epidemiology study *Thorax.* 2004;59:286–290.

36. Verver S., van Soolingen D., Borgdorff M. W.. Effect of screening of immigrants on tuberculosis transmission *International Journal of Tuberculosis and Lung Disease.* 2002;6:121–129.

37. Salpeter E. E., Salpeter S. R.. Mathematical model for the epidemiology of tuberculosis, with estimates of the reproductive number and infection-delay function *American Journal of Epidemiology.* 1998;142:398–406.

38. Schwartzman K., Menzies D.. Tuberculosis screening of immigrants to low-prevalence countries *American Journal of Respiratory and Critical Care Medicine.* 2000;161:780–789.

39. Tuberculosis Control Branch . Report on Tuberculosis Contact Investigations in California, 2001 tech. rep.California Department of Health ServicesBerkeley, California 2001.

40. Asch S., Leake B., Anderson R., Gelberg L.. Why do symptomatic patients delay obtaining care for tuberculosis? *American Journal of Respiratory and Critical Care Medicine.* 1998;157:1244–1248.

41. Sherman L. F., Fujiwara P. I., Cook S. V., Bazerman L. B., Frieden T. R.. Patient and health care system delays in the diagnosis and treatment of tuberculosis *International Journal of Tuberculosis and Lung Disease.* 1999;3:1088–1095.

42. Verver S., Bwire R., Borgdorff M. W.. Screening for pulmonary tuberculosis among

immigrants: estimated effect on severity of disease and duration of infectiousness *International Journal of Tuberculosis and Lung Disease.* 2001;5:419–425.

43. Verver S., Borgdorff M. W.. Personal communication 2004.

44. Taylor Z., Marks S. M., Ríos Burrows N. M., Weis S. E., Stricof R. L., Miller B.. Causes and costs of hospitalization of tuberculosis patients in the United States *International Journal of Tuberculosis and Lung Disease.* 2000;4:931–939.

45. Taylor Z., Marks S.. Personal communication 1998.

46. Brown R. E., Miller B., Taylor W. R., et al. Health-care expenditures for tuberculosis in the United States *Archives of Internal Medicine.* 1995;155:1595–1600.

47. Sciortino S., Mohle-Boetani J., Royce S. E., Will D., Chin D. P.. B notifications and the detection of tuberculosis among foreign-born recent arrivals in California *International Journal of Tuberculosis and Lung Disease.* 1999;3:778–785.

48. Robinson E. S., Mee C. L.. Nursing 2004 Salary Survey *Nursing.* 2004;34:36–39.

49. Palmer C. S., Miller B., Halpern M. T., Geiter L. J.. A model of the cost-effectiveness of directly observed therapy for treatment of tuberculosis *Journal of Public Health Management Practice.* 1998;4:1–13.

50. Weis S. E., Foresman B., Cook P. E., Matty K. J.. Universal HIV screening at a major metropolitan TB clinic: HIV prevalence and high-risk behaviors among TB patients *American Journal of Public Health.* 1999;89:73–75.

51. Moore R. D., Chaulk C. P., Griffiths R., Cavalcante S., Chaisson R. E.. Cost-effectiveness

of directly observed versus self-administered therapy for tuberculosis *American Journal of Respiratory and Critical Care Medicine.* 1996;154:1013–1019.

52. Dion M., Tousignant P., Bourbeau J., Menzies D., Schwartzman K.. Measurement of health preferences among patients with tuberculous infection and disease *Medical decision making.* 2002;22(Suppl.):S102–S114.

53. Dion M., Tousignant P., Bourbeau J., Menzies D., Schwartzman K.. Feasibility and reliability of health-related quality of life measurements among tuberculosis patients *Quality of Life Research.* 2004;13:653–665.

54. Nguyen C., Taylor Z., Qualls N.. Quality of life estimates for tuberculosis Abstract, 30th IUATLD World Conference on Lung Health, Madrid, Spain 1999.

55. Sackett D. L., Torrance G. W.. The utility of different health states as perceived by the general public *Journal of Chronic Disease.* 1978;31:697–704.

56. Stratton K. R., Durch J. S., Lawrence R. S.. , eds. *Vaccines for the 21st Century. A Tool for Decisionmaking.* Washington, D.C.: National Academy Press 1999.

57. Schechter C. B., Rose D. N., Fahs M. C., Silver A. L.. Tuberculin screening: cost-effectiveness analysis of various testing schedules *American Journal of Preventive Medicine.* 1990;6:167–175.

58. Marchand R., Tousignant P., Chang H.. Cost-effectiveness of screening compared to case-finding approaches to tuberculosis in long-term care facilities for the elderly *International Journal of Epidemiology.* 1999;28:563–570.

59. Catlos E. K., Cantwell M. F., Bhatia G., Gedin S., Lewis J., Mohle-Boetani J. C.. Public

health interventions to encourage TB class A/B1/B2 immigrants to present for TB screening *American Journal of Respiratory and Critical Care Medicine.* 1998;158:1037–1041.

60. Stýblo K., van Geuns H. A., Meijer J.. The yield of active case-finding in persons with inactive pulmonary tuberculosis or fibrotic lesions. A 5-year study in tuberculosis clinics in Amsterdam, Rotterdam, and Utrecht *Tubercle.* 1984;65:237–251.

61. Rose D. N., Schechter C. B., Adler J. J.. Interpretation of the tuberculin skin test *Journal of General Internal Medicine.* 1995;10:635–642.

62. Berkel G. M., Cobelens F. G. J., de Vries G., Draayer-Jansen I. W. E., Borgdorff M. W.. Tuberculin skin test: estimation of positive and negative predictive values from routine data *International Journal of Tuberculosis and Lung Disease.* 2005;9:310–316.

63. Villarino M. E., Burman W., Wang Y. C., et al. Comparable specificity of two commercial tuberculin reagents in persons at low risk for tuberculous infection *Journal of the American Medical Association.* 1999;281:169–171.

64. Menzies R. I., Vissandjee B.. Effect of Bacille Calmette-Guerin vaccination on tuberculin reactivity *American Review of Respiratory Diseases.* 1992;145:621–625.

65. Mason J., Freemantle N., Nazareth I., Eccles M., Haines A., Drummond M.. When is it cost-effective to change the behavior of health professionals? *JAMA.* 2001;286:2988–2992.

66. White M. C., Gournis E., Kawamura M., Menendez E., Tulsky J.. Effect of directly observed preventive therapy for latent tuberculosis infection in San Francisco *International Journal of Tuberculosis and Lung Disease.* 2003;7:30–35.

67. Nolan C. M., Goldberg S. V., Buskin S. E.. Hepatotoxicity associated with isoniazid preventive therapy. A 7-year survey from a public health tuberculosis clinic *JAMA.* 1999;281:1014–1018.

68. Maddrey W. C., Boitnott J. K.. Isoniazid hepatitis *Annals of Internal Medicine.* 1973;79:1–12.

69. Clopper C. J., Pearson E. S.. The use of confidence or fiducial limits illustrated in the case of the binomial *Biometrika.* 1934;26:404–413.

70. Menzies R. I., Dion M.-J., Rabinovitch B., Mannix S., Brassard P., Schwartzmann K.. Treatment completion and costs (in a randomized trial) of 4 months rifampin vs 9 months isoniazid *American Journal of Respiratory and Critical Care Medicine.* 2004;epub.

71. Jasmer R. M., Snyder D. C., Chin D. P., et al. Twelve months of isoniazid compared with four months of isoniazid and rifampin for persons with radiographic evidence of previous tuberculosis *American Journal of Respiratory and Critical Care Medicine.* 2000;162:1648–1652.

72. Fountain F. F., Tolley E., Chrisman C. R., Self T. H.. Isoniazid hepatotoxicity associated with treatment of latent tuberculosis infection. A 7-year evaluation from a public health tuberculosis clinic *Chest.* 2005;128:116–123.

73. LoBue P., Moser K.. Use of isoniazid for latent tuberculosis infection in a public health clinic *American Journal of Respiratory and Critical Care Medicine.* 2003;168:443–447.

74. Snider, Jr. D. E., Caras G. J., Koplan J. P.. Preventive therapy with isoniazid. Cost-effectiveness of different durations of therapy *Journal of the American Medical Association.* 1986;255:1579–1583.

75. Salpeter S. R., Sanders G. D., Salpeter E. E., Owens D. K.. Monitored isoniazid prophylaxis for low-risk tuberculin reactors older than 35 years of age: a risk-benefit and cost-effectiveness analysis *Annals of Internal Medicine.* 1997;127:1051–1061.

76. Centers for Disease Control and Prevention . Targeted tuberculin testing and treatment of latent tuberculosis infection *Morbidity and Mortality Weekly Report.* 2000;49:1–51.

77. Iman R. L., Helton J. C., Campbell J. E.. An Approach to Sensitivity Analysis of Computer Models: Part I—Introduction, Input Variable Selection and Preliminary Variable Assessment *Journal of Quality Technology.* 1981;13:174–183.

78. Iman R. L., Helton J. C., Campbell J. E.. An Approach to Sensitivity Analysis of Computer Models: Part II—Ranking of Input Variables, Response Surface Validation, Distribution Effect, and Technique Synopsis Variable Assessment *Journal of Quality Technology.* 1981;13:232–240.

79. Blower S. M., Dowlatabadi H.. Sensitivity and Uncertainty Analysis of Complex Models of Disease Transmission: an HIV model, as an example *International Statistical Review.* 1994;62:229–243.

Table 1: **State of each individual** in the model.

Variable	Interpretation
H_j	Public health status of person j:
	0–if person is eligible for domestic B-notification follow-up
	1–if person will not be actively located and evaluated by a health department
	2–if person j could be sought for evaluation as a recent close contact of an active case of tuberculosis.
B_j	Tuberculosis status of person j:
	0–person is undiseased and has no modeled risk of progression
	1–person is a fast-progressing recently infected individual, but does not have disease
	2–person is latently infected (ATS class 2), but not a fast-progressing recently infected individual
	3–person is in ATS class 4, and eligible for LTBI therapy; (i.e., excluding adequately treated former active cases)
	4–smear-negative active disease, slowly progressing
	5–smear-positive active disease, was slowly progressing
	6–smear-negative active disease, fast progressing
	7–smear-positive active disease, was fast progressing
C_j	approximately, the true ATS class of person j (derived from B_j)
	0–when $B_j = 0$
	2–when $B_j = 2$ or $B_j = 3$
	3–when $B_j = 4$, $B_j = 5$, $B_j = 6$, or $B_j = 7$
	4–when $B_j = 3$
A_j	Age of person j, years
U_j	Undiagnosed indicator; 1 if individual j has ever been diagnosed, 0 otherwise
D_j	Dead indicator; 1 if person j is not alive
O_j	Traces infection to source in original cohort; see text
E_j	Ethnodemographic grouping; see text
S_j	Indexes source of infection; see text
G_j	1 if the individual is a TB4 eligible for treatment, 0 otherwise
θ_j	Time of last tuberculosis status change

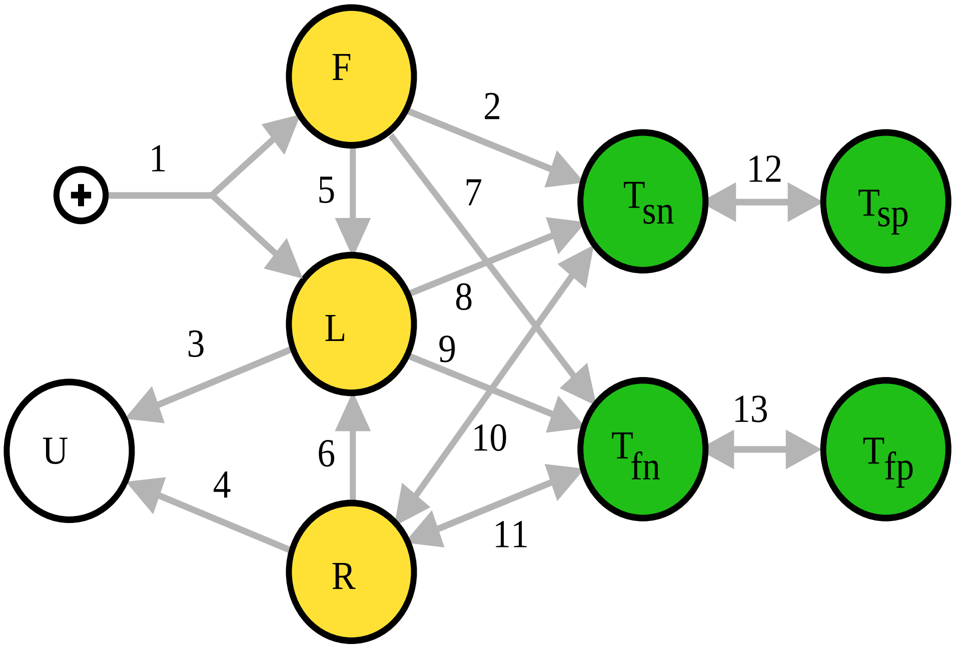

Figure 1. Simplified system diagram for tuberculosis-related classifications of individuals in the cost-effectiveness model. State classifications are indicated by circles; transitions by numbered arrows. Key: 1: new infection (only for close contacts of active cases), 2,7,8,9,10,11: progression to active disease, 3,4: elimination of risk of progression from latently-infected individuals, 5,6: transition to lower risk of progression, 10,11: reverse arrows indicate self-healing of smear-negative active disease, 12,13: progression to smear-positive active disease. Not shown: background mortality from all states, tuberculosis mortality, and diagnosis of active disease. This figure applies to individuals not being sought for evaluation ($H_j = 1$), individuals being sought for domestic B-notification follow-up ($H_j = 0$), and close contacts of active cases if the contact is being sought for evaluation ($H_j = 2$). We assume new infections only occur (arrow 1) when $H_j = 2$. Background and tuberculosis-specific mortality are not indicated in the diagram.

Table 2: **Active events.** In this table, we list all the (possible) events of the model, together with the state conditions under which the event is active (possible). For all events involving person j, the person j must be alive for the event to be active (possible), i.e. $D_j = 0$; for brevity, we omit this from the table column.

Event	Interpretation	Conditions under which event is active (possible)
$\mathcal{P}_j$	Progression of LTBI (reactivation)	$1 \leq B_j \leq 3$
$\mathcal{W}_j$	Progression of active disease	$B_j = 4$ or $B_j = 6$
$\mathcal{H}_j$	Self-healing	$B_j = 4$ or $B_j = 6$
$\mathcal{D}_j$	Death due to undiagnosed tuberculosis	$4 \leq B_j \leq 7$
$\mathcal{M}_j$	Death due to unrelated causes	
$\mathcal{G}_j$	Passive diagnosis	$4 \leq B_j \leq 7$
$\mathcal{B}_j$	Domestic B-notification evaluation	$H_j = 0$
$\mathcal{C}_j$	Evaluation during contact investigation	$H_j = 2$
$\mathcal{T}_j$	Transmission	$4 \leq B_j \leq 7$

Table 3: **Percentage of individuals age 15 and older** in five-year age classes who were reported through B-notification, 1999–2002. Of $15,888$ individuals for which data is available for this period, a further four and one half percent were ages 0 to 14. The percentages do not add to 100 percent due to rounding. Source: California Department of Health Services, Tuberculosis Control Branch.

Age Class	15–19	20–24	25–29	30–34	35–39	40–44	45–49	50–54
Percentage	2.3	2.9	3.6	4.7	6.1	6.9	8.2	10.1
Age Class	55–59	60–64	65–69	70–74	75–79	80–84	85–89	90+
Percentage	10.7	12.7	13.3	9.7	5.7	2.1	0.8	0.1

Table 4: **Approximate five-year mortality rates** for four major ethnodemographic classifications, 1999. Source: derived from California Department of Finance life tables.

Age Class	African-American	Asian/Pacific Islander	Caucasian	Hispanic
15–19	0.00433	0.00206	0.00248	0.00274
20–24	0.00738	0.00193	0.00358	0.00361
25–29	0.00708	0.00185	0.00350	0.00354
30–34	0.00814	0.00257	0.00494	0.00416
25–39	0.0125	0.00336	0.00716	0.00572
40–44	0.0210	0.00536	0.0108	0.00859
45–49	0.0332	0.00882	0.0170	0.0134
50–54	0.0463	0.0130	0.0233	0.0190
55–59	0.0656	0.0213	0.0367	0.028
60–64	0.0960	0.0346	0.0580	0.0449
65–69	0.133	0.0512	0.0895	0.076
70–74	0.196	0.0858	0.138	0.112
75–79	0.279	0.1404	0.207	0.1705
80–84	0.382	0.236	0.327	0.267
85–89	0.496	0.390	0.470	0.374
90+	0.685	0.615	0.743	0.606

Table 5: **Fraction of TB cases** dying during therapy or dead at diagnosis for TB cases not known to be AIDS cases reported in California, 1996 through 2000. Note: Column 2 based on 5925 reported smear-positive cases (126 cases with missing information); Column 3 based on 7449 reported smear-negative cases (603 with missing information); Column 4 based on $16,472$ reported cases (1412 with missing information). Source: California Department of Health Services, Tuberculosis Control Branch, Reports of Verified Cases of Tuberculosis.

Age Group	Death during therapy		Dead at diagnosis
	Smear negative	Smear positive	
15–34	0.79%	1.49%	0.39%
35–64	3.26%	6.91%	1.85%
65+	15.16%	24.18%	5.8%

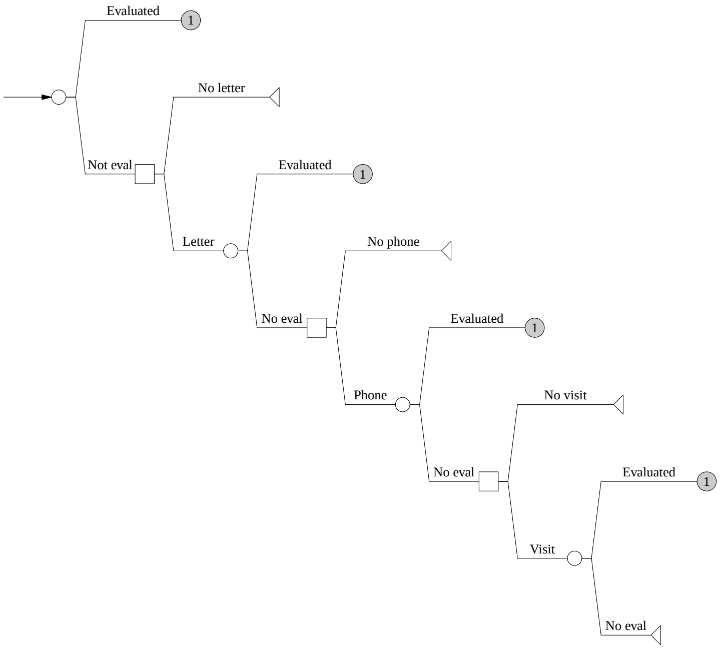

Figure 2. Decision tree for evaluation of B-notification patients based on data from Santa Clara county[59]. Four evaluation effort strategies are illustrated: passive recruitment (no additional effort to recruit subjects), letters ("Letter"), telephone calls ("Phone"), and home visits ("Visit"). The arrow at the left denotes the beginning of the decision tree. For the base case scenario, we assumed no additional evaluation effort (i.e., no cost for letters, phone calls, or home visits) but set the fraction evaluated at the first decision node to the desired value. For simplicity we have omitted the decision whether to invest in a one-time expenditure of funds to improve starting rates; this would only affect the starting rates indicated in the decision tree on Figure 4 (and increase the total cost). The conditional probabilities for the branches are chosen so that the total probabilities of evaluation match those from the Santa Clara study[59]; for the terminal nodes shown, the number of lost QALYs and the number of deaths are zero; the cost is found from the interventions used (i.e., letters, phone, home visits) as given in the cost table in the main text. Circles represent chance nodes, squares represent decision nodes, and triangles represent terminal nodes. The parameters are given in the text. Gray numbered circles indicate links to or from other trees given later.

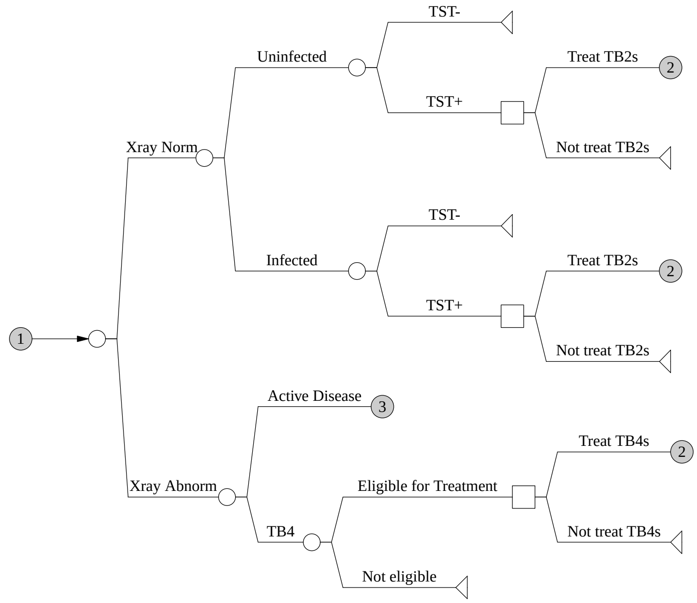

Figure 3. Decision tree for evaluation of B-notification patients including radiographic evaluation and tuberculin skin testing. Symbols are explained in Figure 2. This tree is used to compute the probability of treatment as a function of the sensitivity, specificity, the probability of being uninfected or latently infected with normal chest X-ray (i.e., 1 if $0 \leq B_j \leq 2$ for individual j), the probability of being truly uninfected (given a normal chest X-ray), i.e. 1 if $B_j = 0$ and 0 otherwise, the probability of having active disease given an abnormal chest X-ray (i.e., 1 if $4 \leq B_j \leq 7$ for individual j), and the probability of being eligible for therapy for LTBI given that the person is a TB4 (i.e., 1 if $G_j = 1$ and 0 otherwise).

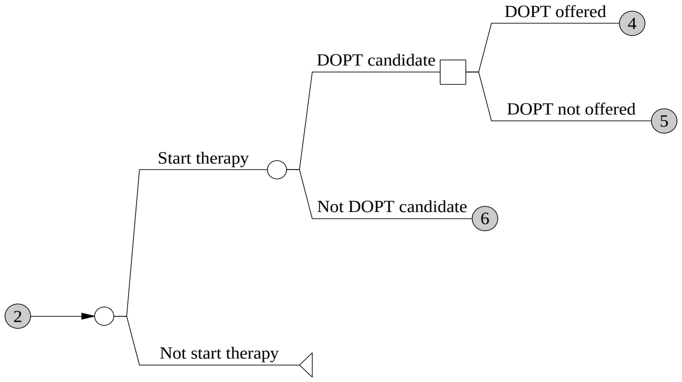

Figure 4. Decision tree for starting LTBI therapy.

Symbols are explained in Figure 2. This tree is used to determine the probability of starting therapy as a function of the probability of starting therapy (which depends on whether or not an intervention has been made to improve starting rates, as described in the text) and the probability that the person is a high-risk person considered a candidate for targeted DOPT (directly observed preventive therapy); these probabilities are described in the text and in Tables 7.

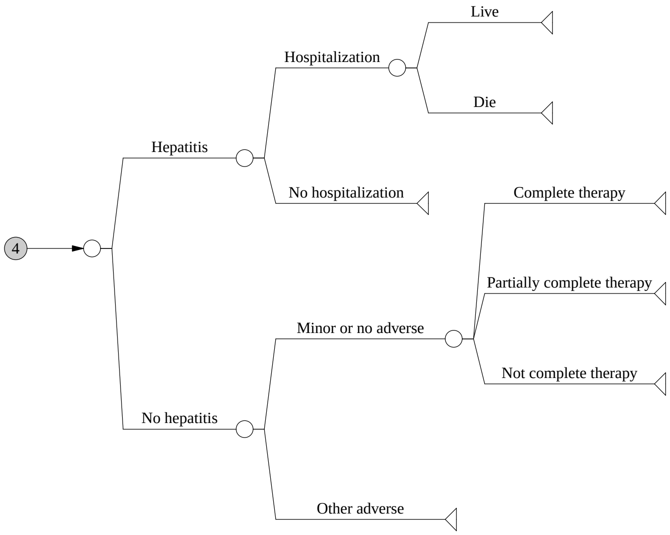

Figure 5. Decision tree for LTBI therapy.

Symbols are explained in Figure 2. This tree is used to determine the probability of death and the probability of completing (or partially completing) therapy, as a function of the probability of INH-related hepatitis, age-dependent, the probability of hospitalization for INH-related hepatitis, the probability of death conditional on hospitalization for INH-related hepatitis, the probability of having minor or no adverse side-effects (important enough to warrant discontinuation), the probability of completing the LTBI-treatment regimen, and the probability of only partially completing the LTBI-treatment regimen. The probability of completion of therapy depends on whether the individual is a high-risk individual and whether or not the person is receiving DOPT (directly observed preventive therapy). See text and Table 7 for numerical values.

Table 6: **Selected natural history parameters.** Note 1: for baseline scenario, we use the more plausible 600 per 10^5; for uncertainty analysis, we chose the highly conservative 430 per 10^5.

Parameter	Value	Range	Reference
Fraction INH-resistant	0.129	0.0645–258	Section 4.10.4
Fraction eligible TB4	0.63	0.55–0.7	3.4
Fraction TB3	0.03	0.0–0.07	3.4
Fraction with normal CXR who are TB2	0.53	0.4–0.6	3.4
Fraction of cases smear-positive	0.075	0–0.15	4.8.3
Fraction of fast smear-positive cases among B-notifications	0.0	const	4.1.2
Undiagnosed			
Death rate, smear negative, undiagnosed	0.02 yr^{-1}	0.0133–0.03 yr^{-1}	4.4
Death rate, smear positive	0.333 yr^{-1}	0.222–0.5 yr^{-1}	4.4
Progression rate, fast smear positive smear negative to smear positive	12 yr^{-1}	6–24	4.1.2
Progression rate, fast smear positive smear positive to smear negative	0.85 yr^{-1}	0.425–1.7 yr^{-1}	4.5
Progression rate, slow smear positive smear negative to smear positive	0.2 yr^{-1}	0.1–0.4 yr^{-1}	4.5
Progression rate, slow smear positive smear positive to smear negative	2 yr^{-1}	1–4 yr^{-1}	4.1.2, 4.5
Self-heal rate, slow, smear negative	0.25 yr^{-1}	0.125–0.5 yr^{-1}	4.3, 4.5
Self-heal rate, fast smear negative	0.0 yr^{-1}	const	4.3, 4.5
Prob. fast disease	0.05	0.02–0.08	4.1.1
Rate of fast disease	1 yr^{-1}	0.8–1.2	4.1.1
Prob. fast smear positive for fast progressor	0.51	0.34–0.765	4.1.2
Prob. fast smear positive for slow progressor	0.51	0.34–0.765	4.1.2
Baseline reactivation rate, TB2	0.00217 yr^{-1}	+/- 10%	4.1.1
Baseline reactivation rate, TB4	0.00430 yr^{-1}; note 1	+/- 10%	4.1.1
Half time, TB2 reactivation rate	13.3 yr	+/- 10%	4.1.1
Half time, TB4 reactivation rate	3.6 yr	+/- 10%	4.1.1
Transmission rate, smear positive	8	3–13	4.7
Transmission rate, smear negative	0.8	0.3–1.3	4.7

Table 7: **Model parameters,** continued. Note 1: 0–1 times the lost QALYs for MDR.

Parameter	Value	Distribution	Reference
Prob. of INH-induced hepatitis			
age 15–34	0.0008	Beta(7,7444)	4.10.5
age 35–64	0.0021	Beta(5,1862)	4.10.5
age 65+	0.0028	Beta(2,359)	4.10.5
Prob. hospitalization from INH-induced hepatitis	0.1	0.05–0.2	4.10.6
Prob. mortality if hospitalized with INH-induced hepatitis	0.125	0.1–0.4	4.10.6
Prob. other adverse events given no INH-induced hepatitis	0.045	0.03–0.06	4.10.7
Health state utility, ultimately fatal TB	0.15	0–0.3	4.9
Health state utility, nonfatal TB	0.45	0.3–0.6	4.9
Hospitalization duration, TB	20 d	15–25 d	4.9
Lost QALYs, TB in treatment	0.15	0.1–0.2	4.9
Lost QALYs, INH prophylaxis, one month	0	const	4.9
Health state utility, INH-induced hepatitis, hospitalized	0.6	0.5–0.7	4.9
Health state utility, nonfatal hepatitis, outpatient	0.735	0.635–0.835	4.9
Utility, other INH-induced adverse events	0.995	0.99–1	4.9
Duration of INH-induced hepatitis hospitalization	8 d	6–10 d	4.10.6
Duration, INH-induced hepatitis, after hospital	1 mo	2–8 wk	4.10.6
Duration, other INH-induced adverse events	15 d	13–18 d	4.10.7
Probability of hospitalization			
Active smear positive	0.35	+/- 10%	4.8.3
Active smear negative	0.081	+/- 10%	4.8.3
Passive smear positive	0.66	+/- 10%	4.8.3
Passive smear negative	0.51	+/- 10%	4.8.3
Prob. cure if LTBI treatment completed	0.7	+/- 10%	4.10.3
Prob. cure if LTBI treatment 6–8 mo.	0.65	+/- 10%	4.10.3
Prob. cure if LTBI treatment 2–5 mo.	0	0	4.10.3
Treatment delay	74 d	64–84 d	4.8.1
TST sensitivity	0.93	0.9–1.0	4.10
TST specificity	0.99	0.9–1.0	4.10
Prob. high risk for noncompletion	0.05	0.0-0.1	4.10.2
Contact finding probability	0.8	0.7–0.9	4.11
Number of screening smears	3	const	4.10.8
Doctor visits, INH-induced hepatitis	3	const	4.10.8
Doctor visits, INH adverse events	1	const	4.10.8
Number of liver function tests, hepatitis	3	const	4.10.8

Table 8: **Uncertainty analysis: incremental cost-effectiveness** of B-notification in California. Each box contains the median value (out of 1000 randomly chosen parameter sets) on the top, and the 5-th and 95-th percentiles on the bottom. Costs are given in 1000s of 2004 US dollars.

Intervention	QALYs saved		Costs incurred		Cases averted		Deaths averted	
Screen all;	8.4		-22.3		0.46		0.22	
treat active cases	0.8	22.2	-388	+335	-0.05	1.70	0.02	0.59
Add treatment	1.6		+15		2.0		0.07	
of TB4s	0.70	3.1	-4.6	+50	1.3	2.8	0.03	0.12
Add treatment	0.39		7.5		0.52		0.02	
of TB2s	-0.01	1.1	1.1	26	0.14	1.1	0.0	0.05
Overall	10.5		6.2		3.1		0.31	
	2.7	25.0	-386	+397	2.0	4.6	0.1	0.7

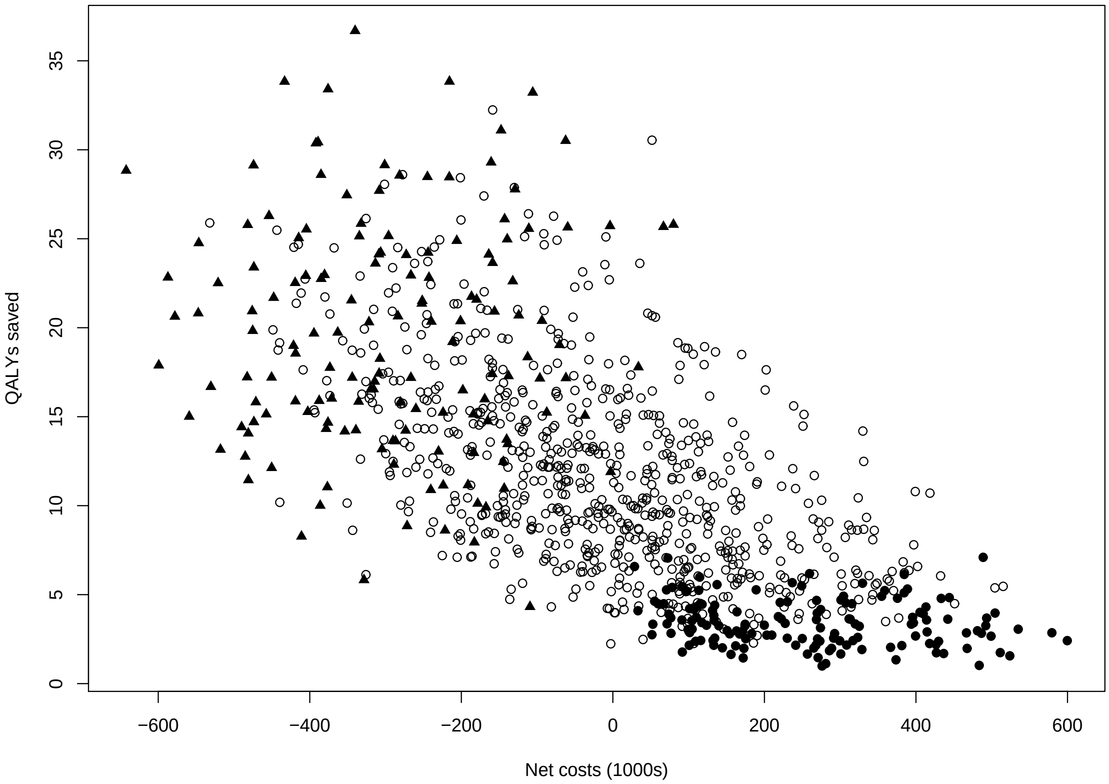

Figure 6. Net costs and QALYs saved overall for B-notification in California, assuming active case finding and treatment of individuals in class TB2 and TB4, for hypothetical cohorts of 1000 people followed for 20 years. Each point (of the 1000 plotted) represents of the average of 1000 replications of the experience of the cohort for a particular randomly chosen scenario. Scenarios with under 1% prevalence of active cases are shown in circles; scenarios with over 6% with black triangles. Negative net costs correspond to savings. Symbols are explained in Figure 2. This tree is used to determine the probability of death and the probability of completing (or partially completing) therapy, as a function of the probability of INH-related hepatitis, age-dependent, the probability of hospitalization for INH-related hepatitis, the probability of death conditional on hospitalization for INH-related hepatitis, the probability of having minor or no adverse side-effects (important enough to warrant discontinuation), the probability of completing the LTBI-treatment regimen, and the probability of only partially completing the LTBI-treatment regimen. The probability of completion of therapy depends on whether the individual is a high-risk individual and whether or not the person is receiving DOPT (directly observed preventive therapy). See text and Table 7 for numerical values.
